# Gender differences in cerebral metabolism for color processing in mice: A PET/MRI Study

**DOI:** 10.1371/journal.pone.0179919

**Published:** 2017-07-19

**Authors:** Philip C. Njemanze, Mathias Kranz, Mario Amend, Jens Hauser, Hans Wehrl, Peter Brust

**Affiliations:** 1 Chidicon Medical Center, Neurocybernetic Flow Laboratory, International Institutes of Advanced Research Training, Owerri, Imo State, Nigeria; 2 Helmholtz-Zentrum Dresden—Rossendorf, Institute of Radiopharmaceutical Cancer Research, Department of Neuroradiopharmaceuticals, Leipzig, Germany; 3 Eberhard Karls University Tübingen, Werner Siemens Imaging Center, Department of Preclinical Imaging and Radiopharmacy, Tübingen, Germany; 4 Helmholtz-Zentrum Dresden—Rossendorf, Department of Mechanical Engineering Dresden, Germany; Radboud Universiteit, NETHERLANDS

## Abstract

**Introduction:**

Color processing is a central component of mammalian vision. Gender-related differences of color processing revealed by non-invasive functional transcranial Doppler ultrasound suggested right hemisphere pattern for blue/yellow chromatic opponency by men, and a left hemisphere pattern by women.

**Materials and Methods:**

The present study measured the accumulation of [^18^F]fluorodeoxyglucose ([^18^F]FDG) in mouse brain using small animal positron emission tomography and magnetic resonance imaging (PET/MRI) with statistical parametric mapping (SPM) during light stimulation with blue and yellow filters compared to darkness condition.

**Results:**

PET revealed a reverse pattern relative to dark condition compared to previous human studies: Male mice presented with left visual cortex dominance for blue through the right eye, while female mice presented with right visual cortex dominance for blue through the left eye. We applied statistical parametric mapping (SPM) to examine gender differences in activated architectonic areas within the orbital and medial prefrontal cortex and related cortical and sub-cortical areas that lead to the striatum, medial thalamus and other brain areas. The metabolic connectivity of the orbital and medial prefrontal cortex evoked by blue stimulation spread through a wide range of brain structures implicated in viscerosensory and visceromotor systems in the left intra-hemispheric regions in male, but in the right-to-left inter-hemispheric regions in female mice. Color functional ocular dominance plasticity was noted in the right eye in male mice but in the left eye in female mice.

**Conclusions:**

This study of color processing in an animal model could be applied in the study of the role of gender differences in brain disease.

## Introduction

Color processing is a central component of mammalian vision. According to the theory developed by Hermann von Helmholtz [1] three types of cone photoreceptors exist, which are classified according to their response to the wavelengths of light striking the retina as short-preferring (blue), middle-preferring (green), and long-preferring (red). The relative strengths of the signals detected by the three types of cones are interpreted by the brain as a visible color. In support of this theory, three wavelength sensitive cone types–short (S) or ‘blue’, middle (M) or ‘green’ and long (L) or ‘red’ have been identified in humans [2]. Parallel to Helmholtz, Ewald Hering proposed the opponent process theory, which states that the visual system interprets color in an antagonistic way: red vs. green, blue vs. yellow, black vs. white [3]. Both theories are now accepted as valid [4] although trichromacy arises at the level of the receptors, and opponent processes arise at the level of retinal ganglion cells and beyond [5]. Thus blue-yellow opponency is reflected in the primate retina by the small bistratified cells classified as "blue-ON, yellow-OFF" and the monostratified giant sparse cells classified as "yellow-ON, blue-OFF" [6] where ON and OFF stands for excitatory and inhibitory signals.

The primary visual pathway originating from the retina comprises the optic nerves, the optic tracts, the dorsal lateral geniculate nuclei (LGN) in the thalamus, the optic radiations and the primary (or striate) and secondary (or extrastriate) visual cortices in the occipital lobes [5]. It has been demonstrated that the retinal ON/OFF organization also applied to neurons at the level of the lateral geniculate nucleus [7], which provide afferent input to the primary visual cortex. Hence, the ON-OFF dichotomy is present in the visual stream from the retina to the first input stage of the primary visual cortex.

In recent years, functional imaging studies have been performed in humans to explore these projections in living subjects with magnetic resonance imaging (MRI) [8–10], high-density diffuse optical tomography [11] or positron emission tomography (PET) [12,13]. Despite this, physiological studies of gender-related differences in color processing in humans are rare. Gender differences in response to red and blue light stimulation have recently been documented using blood oxygen level dependent (BOLD) functional MRI (fMRI) in the human primary visual cortex, where males showed a threefold greater increase to blue light stimulation when compared to females [14]. The differences attributed to blue light, but not red light, were suggested to be related to differences in dopamine function. In accordance to this study our own work using the functional transcranial Doppler (fTCD) technique [15] uncovered differences in blue/yellow color processing between males and females by Fourier analysis of spectral density curves [16–18]. Furthermore, fMRI and diffusion tensor imaging (DTI) were used to explore the activation of the visual cortex associated with color perception in healthy subjects and patients with cerebral dysfunction [19].

Gender-related differences of color vision in primates have been proposed to be genetically predetermined. Thus, while both trichromatic and dichromatic color vision occur among female squirrel monkeys, males appear exclusively dichromatic. It has been proposed that unlike humans, squirrel monkeys have only a single photo pigment locus on the X chromosome [20]. As a classical example, it was established over a hundred years ago from examinations of pedigrees that the common (red-green) color vision defects among humans are inherited as X-chromosome linked recessive traits. It has been proven that, the retinal photo pigments absorbing maximally in the middle to long wavelengths arise from the activity of two genes, both located on the X chromosome [21]. Accordingly, the incidence of defective color vision is much higher in males than in females [22].

There has been much debate about the existence of color vision in other species. According to Walls et al. “within the mammals, color vision is by no means widespread” [23]. However, since Walls’ publication, animal experiments have provided evidence of the presence of color vision in other mammals than primates [24–28]. The photo pigment arrangements in the mouse are similar to that in the rat, and show two types of cone pigments. One cone pigment has a peak sensitivity at about 510 nm, and the other is maximally sensitive in the ultraviolet with a peak at about 370 nm [29]. Color opponency as described above has also been found in mice [30].

Recent developments in imaging technologies enable the investigation of color processing in living animals. PET is an imaging modality that is suitable for brain activation studies [31]. It provides indirect evidence of neuronal activation by measuring hemodynamic and metabolic changes locally within the brain tissue from detection of the radioactive signal of a suitable radiolabelled tracer. Primarily ^15^O-labelled water, a surrogate marker of blood flow, or ^18^F-labelled fluorodeoxyglucose ([^18^F]FDG), a tool to measure regional glucose metabolism, are used for this purpose in humans [32,33] as well as in animals [34,35]. As the local rate of blood flow or energy metabolism is believed to be coupled to neural activity, these surrogate changes are thus used to assess cortical activation and to produce functional brain images. Accordingly, blood flow measurements with PET have been used to investigate color discrimination tasks in rhesus monkeys [36].

Here, we present evidence that PET investigation of the cerebral glucose metabolism using [^18^F]FDG combined with anatomical MRI can be used to study the blue^ON^/yellow^OFF^ and vice versa phenomenon in male and female mice. We intend to identify the gender differences in the innervations of the visual cortical regions in the right and left hemisphere through the right and left eye in male and female mice. The stimuli were designed to include stimulation of all possible retino-geniculo-cortical light transduction pathways to the right and left visual cortex including the V1 ocular dominance columns.

## Materials and methods

### Animals

All animal experiments followed the ‘Principles of laboratory animal care’ (NIH publication no. 85e23, revised 1985) as well as specific national laws approved by the responsible authorities of the state of Saxony, Germany as recommended by the responsible local animal ethics review board (Regierungspräsidium Leipzig, TVV08/13, Germany). All experiments were performed under isoflurane anaesthesia and all efforts were made to minimize pain.

Five male and five female CD-1 mice (10–12 weeks, male = 34 ± 2.8 g; female = 26 ± 1.7 g) (received from the facility of the University of Leipzig, Medizinisch-Experimentelles Zentrum) were housed under a 12 hour light: 12 hour dark cycle at 24°C, 60% humidity in a vented temperature-controlled animal cabinet (HPP110, Memmert GmbH & Co. KG; Schwabach, Germany), with free access to food and water. The same animals were repeatedly imaged with PET on consecutive days without randomization to keep the daytime of measurement (e.g. the glucose/insulin levels) constant. There was no significant change in weight of the animals over the several days of study in male (Day 1 = 34.5 ± 2.8g; Day 2 = 34.4 ± 2.4g; Day 3 = 33.7 ± 2.3g; Day 4 = 34.7 ± 2.3g; Day 5 = 34.3 ± 2.5g; Day 6 = 34.6 ± 2.5g; Day 7 = 34.1 ± 2.6g) and female mice (Day 1 = 25.6 ± 1.7g; Day 2 = 25.4 ± 1.3g; Day 3 = 25.5±1.5g; Day 4 = 25.6 ± 1.2g; Day 5 = 26.4 ± 1.4g; Day 6 = 26.2 ± 1.4g; Day 7 = 26.5 ± 1.3g). The radiotracer ([^18^F]FDG) dose was injected intraperitoneally in male (Day 1 = 12.05 ± 1.23 MBq; Day 2 = 12 ± 0.9 MBq; Day 3 = 11.7 ± 1.2 MBq; Day 4 = 10.6 ± 0.5 MBq; Day 5 = 12.1 ± 1.7 MBq; Day 6 = 10.8 ± 1.2 MBq; Day 7 = 11.9 ± 1 MBq) and female (Day 1 = 12.7 ± 1.23 MBq; Day 2 = 12.7 ± 1.3 MBq; Day 3 = 12.6 ± 0.9 MBq; Day 4 = 13.9 ± 0.7 MBq; Day 5 = 11.4 ± 0.9 MBq; Day 6 = 12.3 ± 1.2 MBq; Day 7 = 12 ± 1.4 MBq) mice and did not vary significantly over the several days of the study. The random blood sugar levels were similar in male (10.1 ± 1.5 mmol/L) and female (7.8 ± 1.8 mmol/L) mice.

At the end of the study the animals were euthanised by cervical dislocation under anesthesia.

### Light stimulation studies

**Fig 1:**
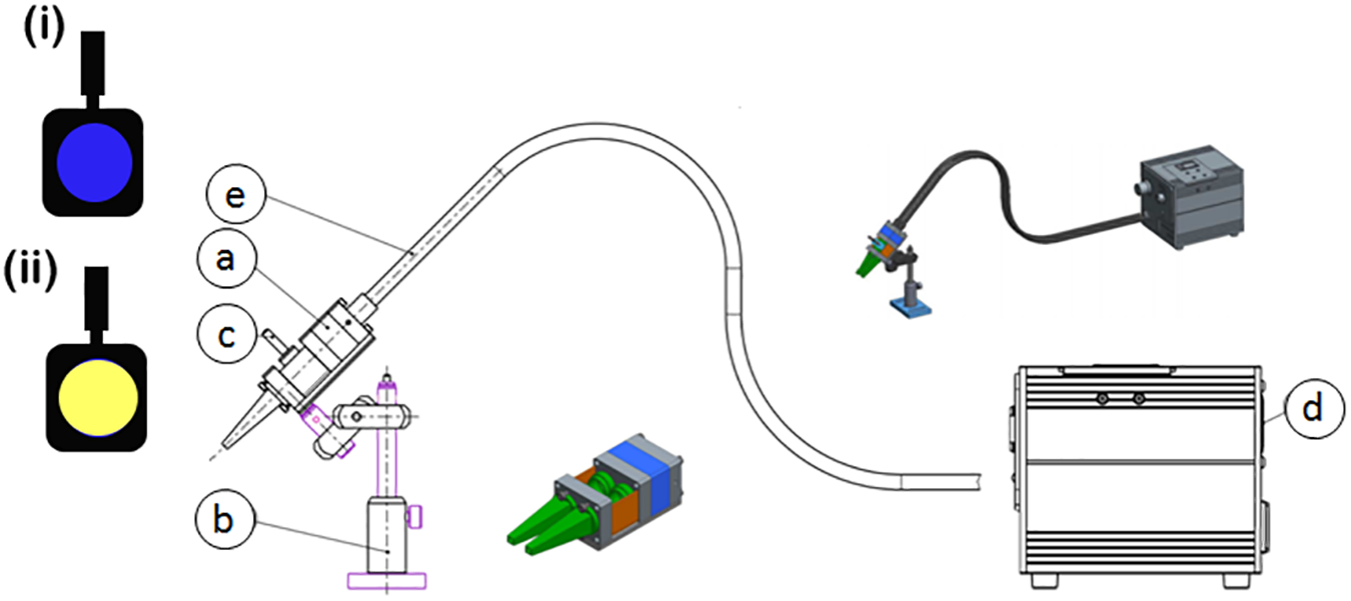
Chromatoscope. Custom-made chromatoscope consisting of the following parts: (a) an optical construction with a connection site for an optical fibre and a white colored reflector shield, (b) a tripod for precise and reproducible positioning above the animal eyes, (c) a filter holder with groove to insert Wratten Kodak filters, Blue (i) and Yellow (ii) as well as an impermeable film for monocular stimulation, (d) a 150 W light source, which produces luminosity with a color temperature of about 3200 K and 20 lumens/watt, (e) an optical fibre, which is guiding the light to the connector at the optical construction (a).

The light stimulation studies were designed to be similar to the ones used in previous human studies [17,18]. The experimental setup included a custom-made animal chromatoscope which is shown in Fig 1. The stimulation device is a double barrel optic placed around both eyes and the nose ridge to separate both visual fields. A white screen illuminated through a light guide (OSL2YFB fibre bundle, Thorlabs Inc., Newton, New Jersey, USA) by a remote light source (Fig 1D) was placed at one end of the optical construction (Fig 1A). The filters were inserted into a groove (Fig 1C) in the right and left visual fields before the screen. The light source (Fig 1D) used was a tungsten coil filament, of a general service lamp ran at a constant 21 V and 150 W, with maximum light output of the bulb of 40,000 foot candles, (∼430,000 lux), power at tip of fibre at a maximum bulb intensity of 1.4 W/m^2^, a color temperature of about 3200 K and 20 lumens/watt (OSL2 High-Intensity Fiber Light Source, Thorabs Inc., Newton, New Jersey, USA). The mouse eye had a fully dilated pupil with a numerical aperture of 0.49, which is twice the numerical aperture of the human eye [37]. The light was presented to the eye over a circular region of ∼24° diameter on the retina. Recordings were made in a laboratory room illuminated by ceiling-mounted fluorescent lamps (150 lux). The light stimulations were accomplished at about the same time of day in the same animal over the several days of study, to maintain synchronization (entrainment) to nature’s cycle of 24 hours. The study in all animals in one day lasted for 6 hours from about 9:30 AM to 3:30 PM, with most male mice studied in the morning hours and female mice in the afternoon hours.

#### Testing color vision in mice using PET/MRI

The spectral absorption curves for the two types of cone pigment found in the mouse retina have λ_max_ as follows: UV (ultraviolet) (360 nm); M (510 nm) [38]. The gelatine Wratten filter 47 has a passband for UV+deep blue. However, most of the UV may not penetrate beyond the anterior of the eye, as a result mainly the deep blue reaches the retina. For the respective color stimulation, the following Wratten filters were used: Deep Blue (No. 47B) with short dominant wavelength of 452.7 nm and Deep Yellow (No. 12) with medium dominant wavelength of 510.7 nm (Kodak Photographic Filters).

The mice were positioned prone in a special mouse bed (heated up to 37°C), with the head fixed to a mouth piece for the anaesthetic gas supply with 1.8% of isoflurane in 40% air and 60% oxygen (Anesthesia unit U-410, AgnTho's AB, Lidingö, Sweden; Gas blender 100, MCQ Instruments, Rome, Italy) while the respiration was monitored for the duration of investigation. For the stimulation, the anesthetized animal was positioned with both eyes open and fixed peeping through the double barrel optic connected to a light source behind the white screen. Mice under narcosis had their eyes open, pupil maximally dilated and did not blink. We employed short duration monocular deprivation for the duration of the stimulation (20 minutes) to excite the contralateral eye. Closure of eye was achieved by covering with 5% dexpanthenol ointment (Bepanthen, Bayer, Germany).

**Fig 2:**
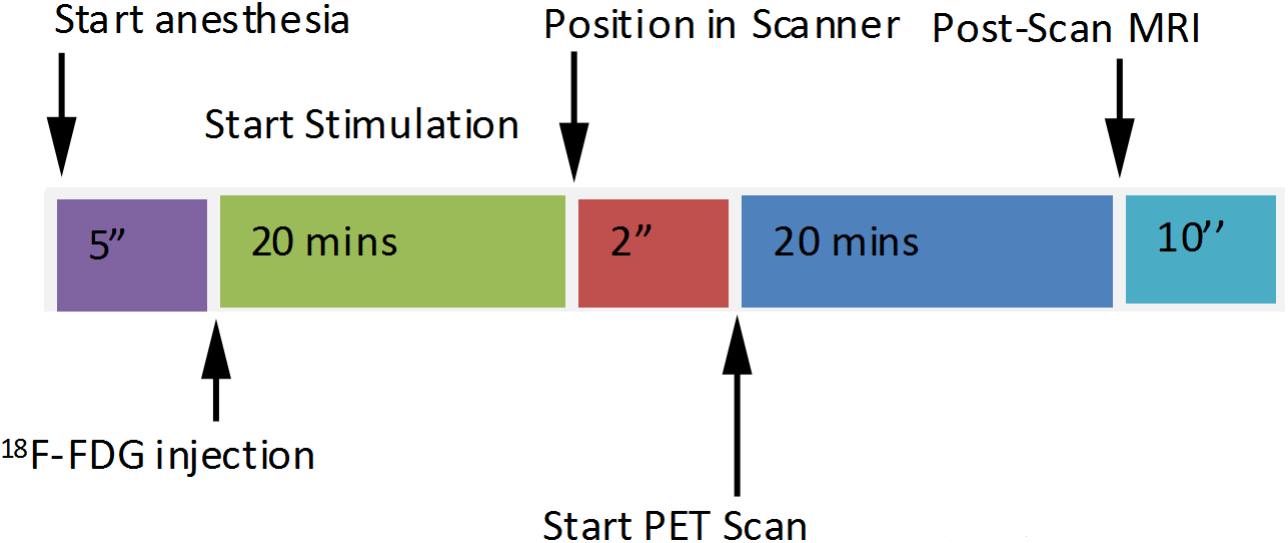
Scan protocol. Timeline for injection, stimulation and data acquisition implemented in the study protocol. (i) 5 min after initiation of anaesthesia about 12 MBq [^18^F]FDG were injected i.p. and immediately followed by 20 min of light stimulation. Warming and respiration monitoring was continued throughout the whole scan protocol. (ii) After stimulation mice were transferred to the PET scanner and it was started for a 20 min whole body scan, (iii) followed by a MRI scan for another 10 min.

The animals received an i.p. injection of 12 ± 1 MBq [^18^F]FDG (Supplier: Prof. M. Patt, Department of Nuclear Medicine, University Hospital Leipzig, Leipzig, Germany) immediately followed by one 20 min stimulation. Subsequently, a whole body PET scan was started for a duration of 20 min, using a preclinical Scanner (nanoScan® PET-MRI, Mediso Medical Imaging Systems, Budapest, Hungary) as shown in the timeline of the scan protocol (Fig 2). Each animal was investigated only once a day while one stimulation and one [^18^F]FDG injection were applied followed by a 24 h recovery period. We established a high-throughput experimentation protocol with time-shift overlaid parallelization. This meant that rather than carrying out single experiments in one animal after another, we overlaid several tasks with start of stimulation in one animal preceding the other by about 25 minutes, therefore shortening the overall time for experiments.

The following seven stimulation conditions were used:

- dark: both eyes (1) closed (dark)
- light: left (2) or right (3) eye open and subjected to standard light source (short: LightL, LightR)
- blue: left (4) or right (5) eye open and subjected to standard light source with blue filter (short: BlueL, BlueR)
- yellow: left (6) or right (7) eye open and subjected to standard light source with yellow filter (short: YellowL, YellowR)

#### PET and MRI data acquisition and analysis

Each PET image was corrected for random coincidences, dead time, scatter and attenuation, based on a whole body MRI scan (T1 weighted gradient echo sequence (GRE), T_R_ = 20 ms; T_E_ = 6.4 ms; matrix size: 160x160x62, resolution: 0.04x0.04x0.05 cm, slice thickness: 0.5mm acquisition duration 12 mins) immediately following the PET acquisition. The attenuation correction is based on a 2-tissue segmentation, animal tissue (attenuation of water) and air. Based on this segmentation a material map is calculated with two homogenous regions containing the μ-values (water and air) to calculate the photon attenuation of the mouse [39]. The T1 images from this sequence were also used for the identification of anatomical details. The PET data was collected by a continuous whole body scan during the entire investigation in list-mode (scan duration 20 min). Subsequently, the data was reconstructed into 4 uniform time frames (5 min each). The reconstruction parameters for the list mode data were 3D-ordered subset expectation maximization (OSEM) with 4 iterations and 6 subsets, energy window: 400–600 keV, coincidence mode: 1–5, ring difference 81.

**Fig 3:**
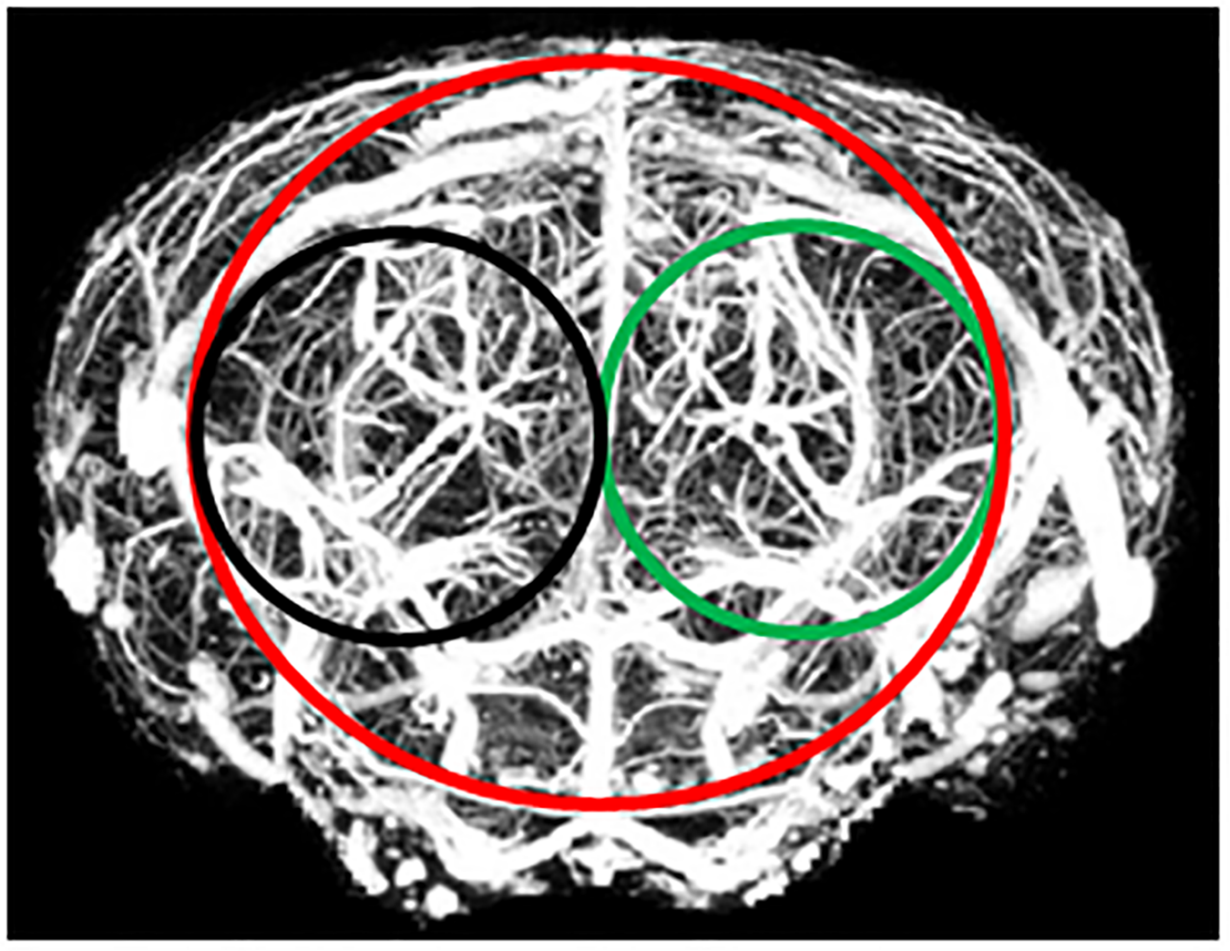
VOI setup in mouse brain. The micro-CT image [40] in coronal view is showing the arterial tree of the middle and posterior cerebral arteries including the cortical and ganglionic branches in the mouse brain. The figure illustrates the VOIs used for data analysis in relation to the brain vessel system, red: Ctx, black: visCtxL and green: visCtxR. [with permission].

The coregistration of the PET/MR data, the delineation of the volume of interest (VOI), and the data analysis were performed by two observers with ROVER (ABX advanced biochemical compounds, Radeberg, Germany, v.2.1.15). First, the PET images were manually coregistered to the respective T1 weighted MR data of each animal. Following this step, the right and left hemispheres were identified using the MRI information from the GRE scan. The VOI in the visual cortex with tracer concentration is a sample volume of a cylindrical mask in a space stretching from the primary visual cortex to the extrastriate cortex perfused by both the ganglionic branches (e.g lenticulostriate arteries) and cortical arteries from the main stems of the middle cerebral artery (MCA) and posterior cerebral artery (PCA) [40]. The actual mesh of arterial trees within the area marked as VOI is demonstrated in the micro-CT image (Fig 3) [41].

The contour VOI is defined as a stack of planar, closed polygons called regions-of-interest (ROI). The contours are manually and semi-automatically outlined on the loaded images, and the pixels contained within the contour boundaries are considered for the VOI statistics. The contour vertices coordinates are defined as the (x, y, z) triples, of which the x, y and z offsets are in [mm] from the image origin. For data analysis three separated VOIs were delineated (Fig 3). First, a whole cortex (Ctx) cylindrical mask with (x, y, z) pixel size (20, 20, 20) or (0.6 cm, 0.6 cm, 0.6 cm) was created to cover most arteries of the two parts of this brain area. It was centred at the midline in coronal view of the PET/MRI image. Using the transverse plane for orientation of tracer accumulation, the VOI extends from the ventromedial occipital region through the posterior inferior temporal cortex. Two sub-volume VOIs with mask (x, y, z) pixel size (10, 10, 10) or (0.3 cm, 0.3 cm, 0.3 cm) were placed from the midpoint to the right border (visCtxR) and to the left border (visCtxL) of the Ctx mask.

The VOIs were defined in homologous areas on both sides of the brain in coronal plane (Fig 3). Hemisphere-specific [^18^F]FDG accumulation was expressed as standardized uptake value (SUV) [42]. The SUV is defined as the ratio of the tissue radioactivity concentration *c* (Bq/g) at time point *t*, and the injected activity divided by the body weight. The investigation of specific tracer uptake was performed at four mid-frame time points: 29.5, 34.5, 39.5 and 44.5 min, after the radiotracer injection. The SUV values were obtained over time for group A (males) and group B (females) under the aforementioned color stimulation conditions, for the three brain regions Ctx, visCtxR and visCtxL. A full quantification of cerebral metabolic rate of glucose (CMR_Glc_) would require dedicated kinetic modelling as well as arterial blood sampling. Due to the small blood volume of mice, repeated arterial blood sampling is challenging, furthermore the study design did not allow us to obtain PET data in this study immediately following the injection of [^18^F]FDG. Therefore we refrain from kinetic modelling, but used the SUV values as a surrogate marker for CMR_Glc_

#### Statistical parametric mapping

To achieve a three-dimensional voxel-by-voxel group comparison statistical parametric mapping (SPM) was performed using the software SPM12 (Wellcome Trust Centre for Neuroimaging, London UK, http://www.fil.ion.ucl.ac.uk/spm/). Each PET data set from a single animal and the respective stimulation was added to form an averaged static image from 29.5–44.5 min p.i. (70 static PET images in total, 5 male and 5 female mice per stimulation as shown in Table 1). Subsequently, each image was manually coregistered to a 3D MRI-atlas as available in PMOD v. 3.6 (PMOD Technologies, Zurich, Switzerland). To account for differences based on misalignment between the static PET images and the atlas as well as to limit the effects of inter-individual anatomical variations a smoothing filter was applied with a kernel size of 1.5 mm full width at half maximum in all three of the spatial dimensions (isotropic smoothing). In order to account for global impairment effects in an animal, a proportional scaling was used on each image to scale them to the image’s global mean voxel value [SPM12 Manual page 92, http://www.fil.ion.ucl.ac.uk/spm/doc/manual.pdf]. The design for the statistical analysis was defined into 5 x 5 animal static PET matrices. Subsequently, a voxel-wise two-tailed *t*-test was calculated for female dark vs. stimulation, male dark vs. stimulation and male vs. female. The resulting three-dimensional functional images (T-value maps for *p*<0.05 uncorrected) were superimposed with MRIcron (Neuropsychology Lab, Columbia SC, USA, v.1.40) on the same MRI atlas for anatomical orientation. The brain activation maps were compared regarding the statistical significance of the activated voxels (maxima in the T maps) [34]. In addition, a family-wise error correction was performed. Due to the small group size of our experimental setup no significant findings remained However when we applied a threshold cluster size of 10 active voxels or even 20 active voxels, we still observed significantly activated areas indicating that the observed activations are real effects, but may not entirely preclude false positives. Nevertheless care was taken when interpreting results by considering only multiple voxel activations.

**Table 1:** SUV values (mean ± SD) in the whole cortex, right and left visual cortex in male and female mice, percent changes (Δ %) and significant differences (*t*-test results) between genders after application of [^18^F]FDG and the respective light stimulation.

Stimulation through Eye (R, L)	Brain area	Male	Female	Δ %	t-value	p <
*Dark	**Ctx**	1.1±0.1	0.95±0.1	15.8%	4.483	0.0001
*Dark	**visCtxR**	1.38±0.16	1.21±0.14	14%	3.421	0.01
*Dark	**visCtxL**	1.33±0.2	1.21±0.12	9.9%	2.318	0.05
**LightR**	**Ctx**	1.08±0.25	0.92±0.15	17.4%	2.400	0.05
**LightR**	**visCtxR**	1.29±0.37	1.07±0.12	21.7%	2.388	0.05
**LightR**	**visCtxL**	1.27±0.37	1.06±0.1	19.8%	2.408	0.05
**LightL**	**Ctx**	1.08±0.16	1.05±0.17	2.9%	0.634	NS
**LightL**	**visCtxR**	1.36±0.24	1.28±0.24	6.3%	-0.996	NS
**LightL**	**visCtxL**	1.36±0.24	1.25±0.25	8.8%	1.383	NS
**BlueR**	**Ctx**	1.16±0.09	1.01±0.1	14.9%	5.045	0.0001
**BlueR**	**visCtxR**	1.47±0.1	1.26±0.16	16.6%	4.974	0.0001
**BlueR**	**visCtxL**	1.53±0.08	1.28±0.13	19.5%	7.431	0.0001
**BlueL**	**Ctx**	1.02±0.1	1.11±0.1	-8.1%	-2.955	0.01
**BlueL**	**visCtxR**	1.28±0.15	1.34±0.08	-4.5%	-1.748	NS
**BlueL**	**visCtxL**	1.25±0.11	1.3±0.11	-3.8%	-1.315	NS
**YellowR**	**Ctx**	1.13±0.08	0.96±0.05	17.7%	8.143	0.0001
**YellowR**	**visCtxR**	1.47±0.12	1.24±0.09	18.5%	6.684	0.0001
**YellowR**	**visCtxL**	1.45±0.1	1.18±0.09	22.9%	8.632	0.0001
**YellowL**	**Ctx**	1.03±0.1	1.0±0.13	2.9%	0.751	NS
**YellowL**	**visCtxR**	1.25±0.16	1.26±0.13	-0.8%%	-0.065	NS
**YellowL**	**visCtxL**	1.23±0.18	1.27±0.14	-3.1%	-0.774	NS

*Dark in both eyes.

#### Data Statistics

All analyses were performed using the software packages Statistica for Windows (Dell, Aliso Viejo, CA, USA) and SPSS Version 20 (IBM, Armonk, NY, USA). Results are displayed as Mean ± SD. Multivariate analysis of variance (MANOVA) with repeated measures was applied. Furthermore, specific differences between various conditions were analysed using the paired or unpaired *t*-test. The level of significance was set at *p*≤0.05.

## Results

To explore gender differences in color processing in mice, the data measured comprised SUV of [^18^F]FDG, at four times: 29.5, 34.5, 39.5 and 44.5 min, post injection and visual stimulation of 20 min duration. The data was acquired for each of the following seven conditions investigated in anaesthetized male and female mice:

- dark: both eyes covered (short: Dark)
- light: left or right eye open and subjected to standard light source (short: LightL, LightR)
- blue: left or right eye open and subjected to standard light source with blue filter (short: BlueL, BlueR)
- yellow: left or right eye open and subjected to standard light source with yellow filter (short: YellowL, YellowR).

### Gender differences in SUV

Table 1 presents the mean SUV values for male and female mice in Ctx, visCtxR and visCtxL. The results serve to investigate the gender differences in SUV under each light stimulation condition applied in this study. The gender-related differences (in Δ %) show that, during all stimulations through the right eye, the SUV change, in male mice was much higher than in female mice with a maximum effect for YellowR visCtxL. Furthermore, the results of the t-test confirm that these findings are highly significant (p values <0.05) for the right eye stimulation but not for the left eye (except for BlueL Ctx).

Table 2 shows that in male mice, the increase in SUV was highest with blue light stimulation through the right eye in the left visual cortex (BlueR visCtxL), which was above baseline dark condition and significantly higher than in the right visual cortex (BlueR visCtxR), *p* < 0.05. On the other hand, in female mice compared to dark baseline condition, the SUV was highest for blue stimulation through the left eye in the right visual cortex (BlueL visCtxR), which was significantly higher than in the left visual cortex (BlueL visCtxL), *p* < 0.01. Note that, due to the overall higher levels of SUV in male mice compared to female mice (see Table 1), the increase in SUV during blue stimulation through the left eye in the right visual cortex (BlueL visCtxR) showed a tendency but was not significantly higher than that in male mice.

**Table 2:** [^18^F]FDG SUV in the right and left visual cortex during study conditions and percent changes (Δ %) from dark condition observed in male and female mice.

Stimula-tion/Eye	visCtxR	Δ %	p <	p-value Eyes	visCtxL	Δ %	p <	p-value Eyes	*p-value visCtx
Male Mice									
DarkR	1.38±16				1.33±0.2				
DarkL	1.38±16			NS	1.33±0.2				
LightR	1.29±0.37	-6.5%	NS		1.27±0.36	-4.5%	NS		NS
LightL	1.36±0.24	-1.4%	NS	NS	1.36±0.24	2.3%	NS	NS	NS
BlueR	1.47±0.1	6.5%	NS		1.53±0.08	15%	0.01		0.05
BlueL	1.28±0.15	-7.2%	NS	0.0001	1.25±0.1	-6%	NS	0.0001	NS
YellowR	1.47±0.12	6.5%	0.05		1.45±0.1	9%	0.01		NS
YellowL	1.25±0.16	-9.4%	0.01	0.0001	1.23±0.2	-7.5%	0.01	0.0001	0.05
Female Mice									
DarkR	1.21±0.14				1.21±0.12				
DarkL	1.21±0.14			NS	1.21±0.12				
LightR	1.07±0.11	-11.6%	0.001		1.06±0.1	-12.4%	0.0001		NS
LightL	1.29±0.24	6.6%	NS	0.0001	1.25±0.25	3.3%	NS	0.001	NS
BlueR	1.26±0.16	4.1%	NS		1.28±0.13	5.8%	0.05		NS
BlueL	1.34±0.08	10.7%	0.01	NS	1.3±0.11	7.4%	0.05		0.01
YellowR	1.24±0.09	2.5%	NS		1.18±0.09	-2.5%	NS		0.01
YellowL	1.26±0.13	4.1%	NS	NS	1.27±0.14	5%	NS	0.0001	NS

R, right eye; L, left eye; visCtx, visual cortex

* side-to-side differences for each condition

**Fig 4:**
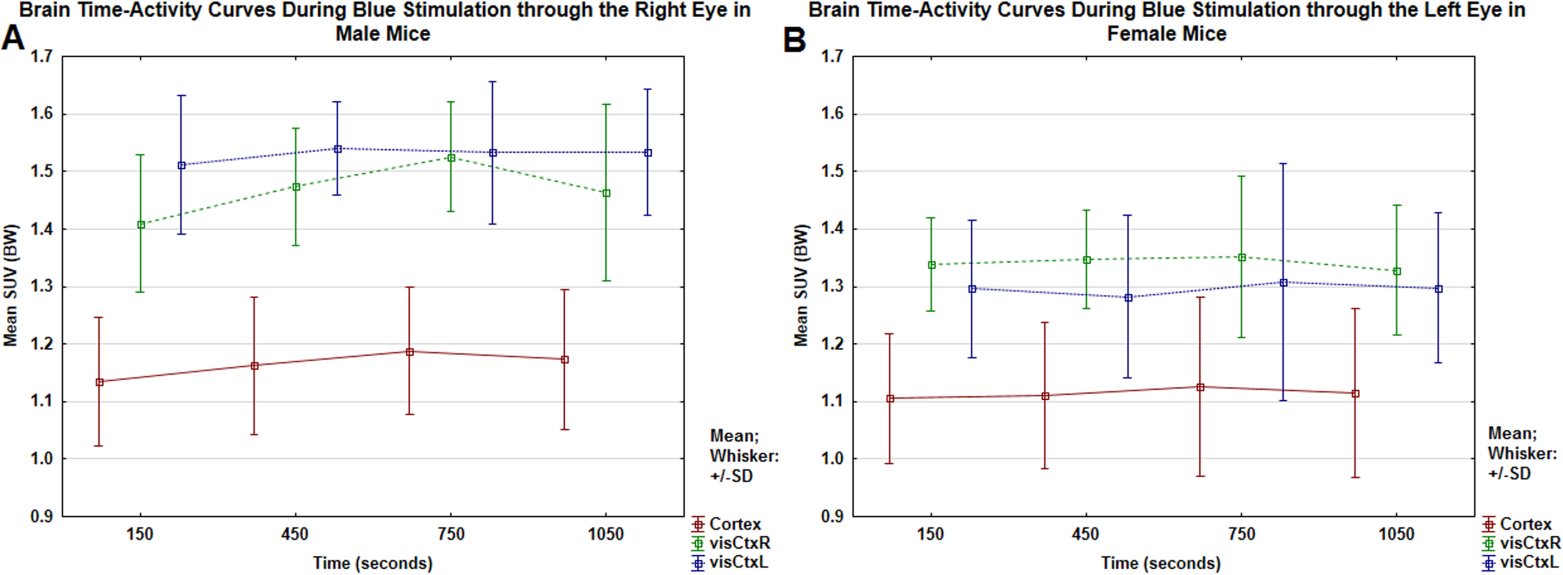
Brain time-activity curves. The time-activity curves show that, in male mice, the mean SUV uptake during blue stimulation through the right eye was higher in the left visual cortex (BlueR visCtxL) than in the right (BlueR visCtxR) (Fig 4A). On the other hand, in female mice, the mean SUV uptake during blue stimulation through the left eye was higher in the right visual cortex (BlueL visCtxR) than in the left (BlueL visCtxL) (Fig 4B). The times of measurements are identical (150 s, 450 s, 750 s, and 1050 s) for all three regions.

Furthermore, significant opponent responses revealed as inhibitory decrements in SUV values would be expected with yellow light stimulation in the visual cortex where there was activation with blue light stimulation. In male mice, yellow light stimulation through the left eye evoked inhibitory decrements in SUV values in the left (YellowL visCtxL) and right visual cortex (YellowL visCtxR). Conversely, in female mice, there were no responses to yellow light stimulation. The most profound findings during blue light stimulation through the right eye in male mice and through the left eye in female mice, agree with trends visualized in brain time-activity curves in Fig 4. Fig 4A shows that in male mice, there was higher activity during blue light stimulation through the right eye in the left visual cortex (BlueR visCtxL) than in the right visual cortex (BlueR visCtxR). On the other hand, in female mice, there was higher activity during blue light stimulation through the left eye in the right visual cortex (BlueL visCtxR) than in the left visual cortex (BlueL visCtxL) (Fig 4B). Overall, the time-activity curves for cortex, visCtxR, visCtxL were higher in male mice than in female mice (Fig 4).

### Interaction effects of study conditions

**Fig 5:**
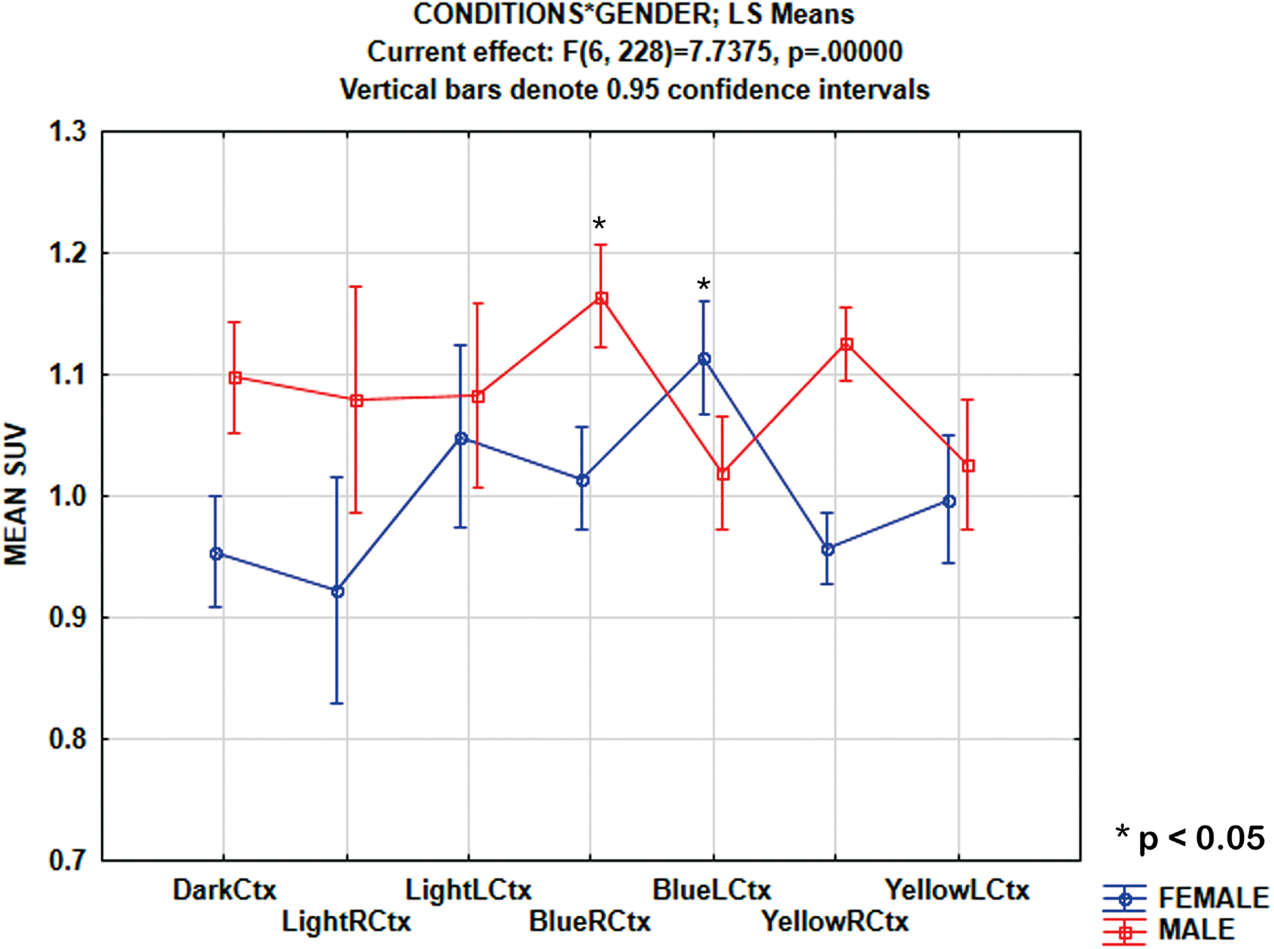
MANOVA results from gender and stimulation comparison. The conditions-gender interactions show that the SUV in the cortical area (Ctx) for dark, light, and yellow is higher in male than female mice. In male mice, the SUV in BlueR Ctx was higher than in female mice. Conversely, in female mice, SUV in BlueL Ctx was higher than in male mice.

To analyse the gender-related differences in SUV in the cortical area (Ctx) during all conditions, a MANOVA with repeated measures of [18F]FDG SUV with a 7 x 2 design: seven levels of conditions (Dark, LightR, LightL, BlueR, BlueL, YellowR, YellowL) and two levels of gender (male, female) was performed. There was a main effect of the gender, F(1,38) = 11.4, MS = 0.49, p < 0.01 and of the conditions, F(6,228) = 3.3, MS = 0.0418, p < 0.01. Furthermore, there was a condition-gender interaction, F(6,228) = 7.7, MS = 0.097, p < 0.0001. The conditions-gender interactions are summarized in Fig 5, showing that the SUV in the cortical area (Ctx) for Dark, Light and Yellow conditions were higher in male than in female mice. Applying one-way ANOVA, it revealed that during blue stimulation through the right eye in the cortical area (BlueR Ctx), the SUV was higher in male than in female mice, *F*(1,38) = 25.5, *MS* = 0.226, *p* < 0.0001.

However, the converse effect occurred during blue stimulation through the left eye (BlueL Ctx), where SUV was higher in female than male mice, *F*(1,38) = 8.73, *MS* = 0.091, *p* < 0.01.

**Fig 6:**
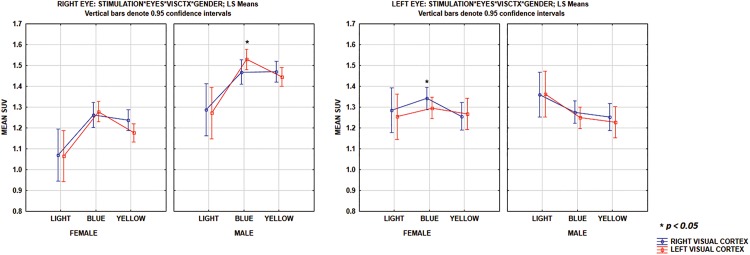
MANOVA results from visCtx and gender comparison. The stimulations-eyes-visual cortex interaction shows that during blue stimulation through the right eye, there was higher SUV in the left visual cortex (BlueR visCtxL) in male mice. Conversely, in female mice, during blue stimulation through the left eye, the right visual cortex (BlueLvisCtxR) showed higher SUV than the left visual cortex (BlueL visCtxL).

To examine the effects of stimulations on the right and left visual cortex (visCtxR and visCtxL) in male and female mice, an MANOVA was performed with repeated measures of [^18^F]FDG SUV with a 3 x 2 x 2 x 2 design: three levels of stimulations (light, blue, yellow), two levels of eyes (right, left), two levels of visual cortex (visCtxR, visCtxL) and two levels of gender (male, female) as the grouping variable (Fig 6). There was a main effect of the gender, *F*(1,38) = 15.3, MS = 1.66, *p* < 0.001 and the stimulations, *F*(2,76) = 3.9, *MS* = 0.35, *p* < 0.05.

To examine the specific effects of the different conditions, a planned *t*-test was performed and the results summarized in Table 2. There was no significant difference between dark/light through the right and left eyes on the right visual cortex and left visual cortex in male mice. Furthermore in male mice, the highest increment was found for blue stimulation through the right eye in the left visual cortex (BlueR visCtxL), with 15%, *p* < 0.01. The highest increase for yellow stimulation was through the right eye in the left visual cortex (YellowR visCtxL), by 9%, *p* < 0.01.

Stimulation with yellow light through the left eye caused in the left visual cortex (YellowL visCtxL) a decrease of the SUV by -7.5%, *p* < 0.01, in male mice. In the right visual cortex of male mice, stimulation with yellow light through the right eye (YellowR visCtxR) caused a modest increase by 6.5%, *p* < 0.05. On the other hand, stimulation of the right visual cortex with yellow through the left eye (YellowL visCtxR) decreased SUV by -9.4%, *p* < 0.01, in male mice.

In female mice, light stimulation through the right eye but not left, decreased the SUV in the right visual cortex (LightR visCtxR), by -11.6%, *p* < 0.01, and left visual cortex (LightR visCtxL), by -12.4%, *p* < 0.0001. Color stimulation caused the highest increment for blue through the left eye in the right visual cortex (BlueL visCtxR), by 10.7%, *p* < 0.01. There was a smaller increase for blue through the left eye in the left visual cortex (BlueL visCtxL), by 7.4%, *p* < 0.05. Furthermore, a smaller increment was observed during blue stimulation through the right eye in the left visual cortex (BlueR visCtxL), by 5.8%, *p* < 0.05. There was no change with yellow stimulation.

**Fig 7:**
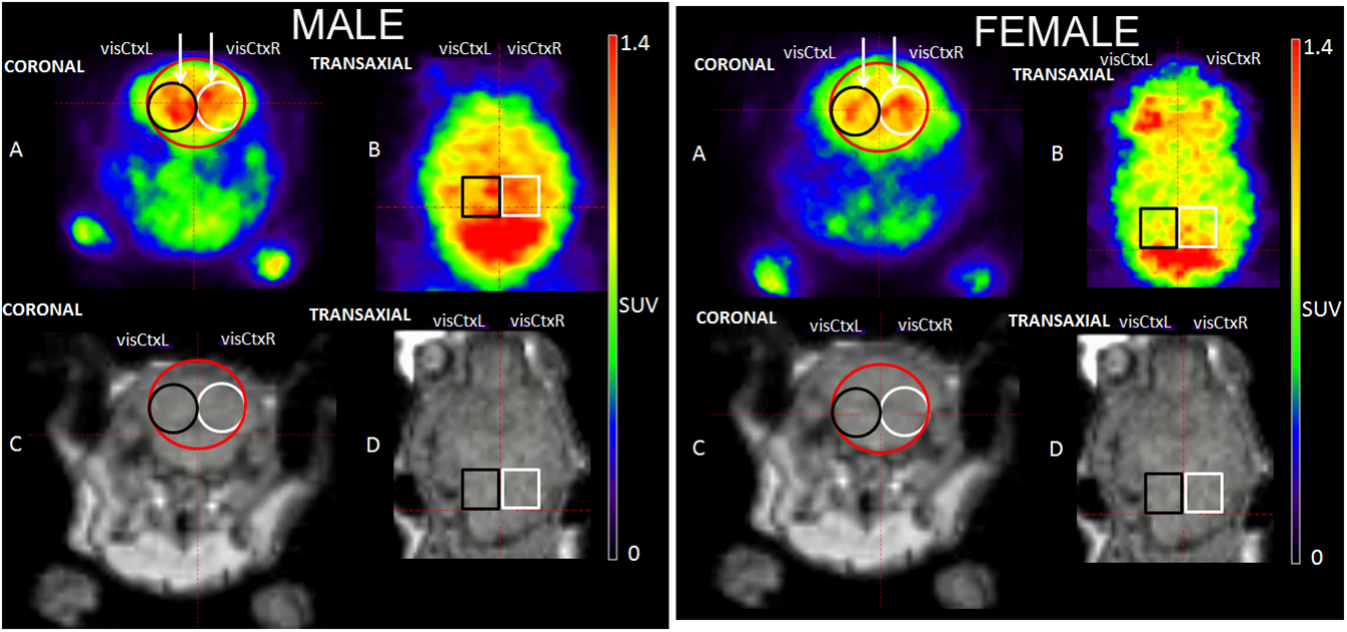
PET/MR images and VOI positioning. [^18^F]FDG brain PET (A),(B) and MR (C),(D) images of a male and a female CD-1 mouse in coronal (A),(C), and transverse (B), (D) views. The color scales show regions of highest SUV in red. T1 weighted MR images were obtained by a gradient echo sequence with TE = 6.4 ms and TR = 20 ms in coronal (C) and transverse (D) views. For the analysis of the visual cortex three cylindrical VOIs were created. The large x, y, z (20, 20, 20) pixel mask (red color), Ctx, contains most of the arterial branches (MCA and PCA) of the two parts of this brain area. The two small VOIs with the dimension of x, y, z (10, 10, 10) pixel (black: visCtxL, white: visCtxR) are defined in homologues areas inside the visual cortex. In male mice, during blue stimulation through the right eye, the higher SUV values occurred in the left visual cortex (black: BlueR visCtxL), while in female mice, during blue stimulation through the left eye, the higher SUV values were in the right visual cortex (white: BlueL visCtxR).

Fig 7, shows the [^18^F]FDG brain PET and MR images (gradient echo sequence with TE = 6.4 ms and TR = 20 ms) of areas of the highest SUV in the visual cortex during blue stimulation through the right eye in male and left eye in female CD-1 mouse in coronal (A), (C), and transverse (B), (D) views. The color scales show regions of highest SUV in red. Note that the brain area with intense tracer accumulation (Fig 7B) behind the VOI is the cerebellum. Furthermore, it was consistently observed that, light stimulation increased the tracer uptake in the stimulated eye compared to the unstimulated eye.

### Light stimulation induced change in glucose consumption

**Fig 8:**
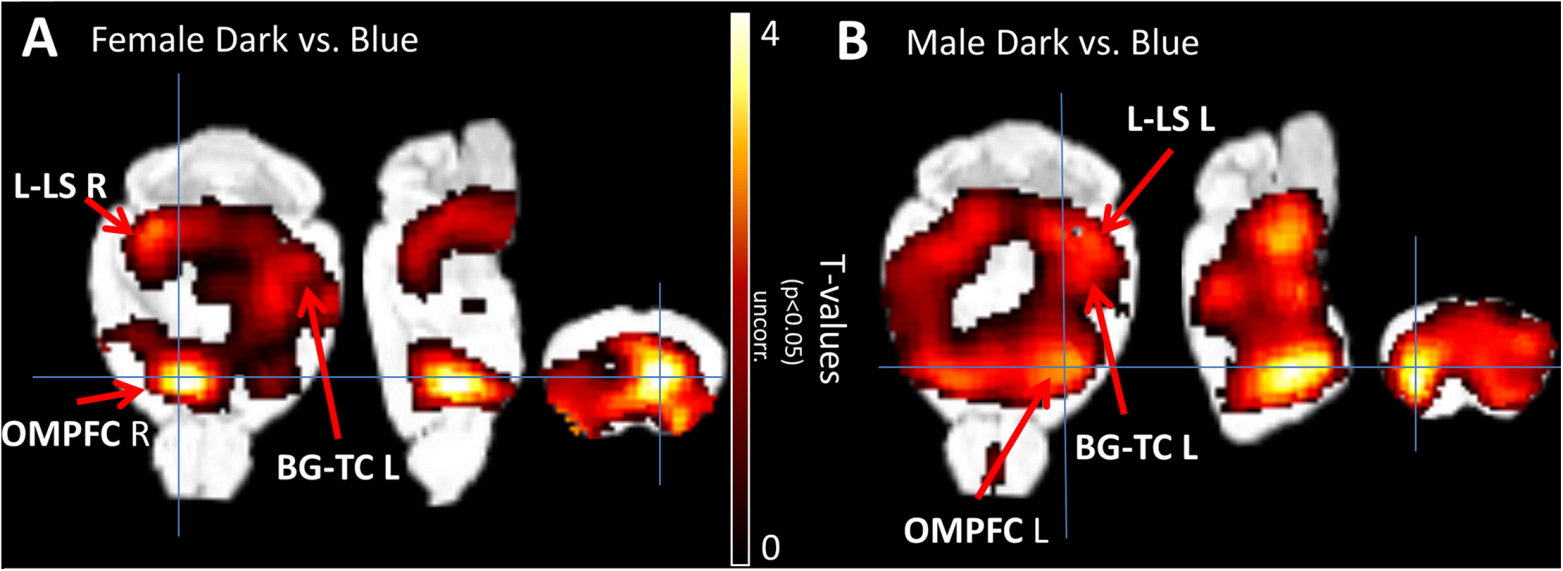
Statistical parametric maps showing *t*-test results, as demonstrated in Tables 1 and 2, of blue stimulation compared to dark conditions. The figure is showing the colored overlay of T-values with an MRI atlas as published [43]. The *t-*test (values for *p*<0.05 uncorrected, are shown) was applied pixel-wise between dark and blue stimulation in male or female mice. The anatomical assignment of the ROIs at pixel level are marked by red arrows. The figure shows the estimated T-values in transaxial, sagittal and coronal views of dark versus blue stimulation calculations with SPM in female (Fig 8A) and male (Fig 8B) mice, respectively. Male mice show a clear activation in visCtxL and visCtxR for dark condition in contrast to blue light. The significant differences in glucose uptake evoked by blue stimulation in female mice showed contralateral interregional activation of right orbital and medial prefrontal cortex (OMPFC R), left basal ganglia-thalamic nuclei (BG-TC L), right limbic/limbic-related structures (L-LS R) and right visual cortex. On the other hand, in male mice, during blue stimulation there was greater intraregional activation of left orbital and medial prefrontal cortex (OMPFC L), left basal ganglia-thalamic nuclei (BG-TC L), left limbic/limbic-related structures (L-LS L) and left visual cortex.

Fig 8A and 8B graphically illustrates the statistical data in Tables 1 and 2. Furthermore, it shows that, blue stimulation activated cortical areas of the orbital and medial prefrontal cortex (OMPFC), a complex region containing agranular, dysgranular, and granular regions which interact with the basal ganglia-thalamic circuits, limbic and limbic-related structures. In female mice, the right OMPFC showed interregional significant differences of metabolic activities with the contralateral left basal ganglia-thalamic circuits and with limbic and limbic-related structures predominantly in the right hemisphere. Conversely, in male mice, the left OMPFC showed greater intraregional metabolic activities with the left basal ganglia-thalamic circuits and with limbic and limbic-related structures predominantly to the left hemisphere.

## Discussion

The study presents novel findings in mice showing gender-related effects due to light stimulation while the setup is based on a design developed for human studies [16–18]. The device was designed to evaluate the brain response to monocular stimulation with light of different wavelengths compared to dark and white light conditions. We examined the changes relative to dark condition, even though, the sensation of black is not the same as absence of light, which is the central tenet of the Herings hypothesis [44]. The device is fully controlled by an observer and totally precludes subject movement artefacts in conscious humans or anaesthetized animals. The experimental design implemented a steady light source as was used in humans rather than flashes of light. The latter is important, since the derived response can only be related to wavelength effects of the stimuli rather than the alternating effects of light-dark condition with flashes of light which cause circadian resetting in mice [45]. Color stimulation was accomplished using optically homogenous filters with specific spectral characteristics [46] with steady light, which has demonstrated consistent results in human studies [16,18]. Simple visual perception of color cardboard sheets in constant room light was effective in demonstrating the effects of colors on the human brain [47]. In this study, the stimulation was directed to color processing neurons, receiving input from only one eye at a time. These neurons are known to be grouped together within the same area of the striate cortex, extending from the upper to lower cortical layers, and are referred to as ocular dominance column (or blobs) [48]. On the other hand, those receiving inputs from both eyes are called hypercolumn [48].

The study design has some drawbacks. One important aspect is the small sample size of the experimental groups, which limits the statistical power of data analysis. Accordingly, family-wise error correction did not reveal significances. However, using the uncorrected data and increasing the threshold cluster size in SPM (showing only areas where at least 10 or 20 adjacent voxels are active) did not change the significant results shown in Fig 8 indicating that the observed activations are real. Our recent study [34] has shown the feasibility of brain activation and FDG-PET imaging studies using a similar sample size.

Furthermore, multiple measurements were performed on each animal which limits the effect of a single outlier on the means. There was no randomization within the block design for time of day and study since it was more important to maintain synchronization with the circadian nature’s cycle of 24 hours. Even though it is not known whether there could have been any differences introduced by the procedural design that male mice were studied in the morning hours and female mice in the afternoon hours, a larger sample size could have provided the possibility for randomization that included the opposite groups. Despite these limitations this initial pilot study provides important insights into visual activation in mice and allows improving the study design in the future.

Very recently, real-time imaging of brain activity under visual stimulation in freely moving rats using functional ultrasound has been performed [49]. Long (7 s) or short (2 s) flashes of blue light were randomly presented to the rats in their cages. Both stimuli elicited a significant increase (5–10%) of the cerebral blood volume signal in the lateral geniculate nucleus. We expected that movement artefacts and highly variable stress-related signal in fully awake animals would overlay the visual responses, which would strongly influence the statistical analysis. Our setup allows a detailed investigation of color processing using [^18^F]FDG as a marker of overall brain metabolic activity during the time of measurement [50]. We have administered [^18^F]FDG intraperitoneally immediately before the visual stimulation and started the PET measurement immediately thereafter. Therefore our measurement of the SUV of [^18^F]FDG reflects CMR_Glc_ during visual stimulation. As previously has been shown, intraperitoneal injection is a valid alternative route, providing pharmacokinetic data equivalent to data from tail-vein injection for small-animal [^18^F]FDG PET [51]. The SUVs of [^18^F]FDG (between 0.9 and 1.5) which we measured in our study in CD-1 mice are similar to the data which was recently reported in C57BL/6 mice under similar conditions [51,52]. The isoflurane anaesthesia which we have chosen was found to be suitable for small animal [^18^F]FDG PET imaging [53]. Others have suggested a novel method to track changes in FDG metabolism dynamically, with higher temporal resolution than exists to date and within a single session [54]. However, its adaptability and application for our present study design using a steady stimulation may not be feasible.

In male mice we have observed (see Fig 6) a higher SUV in the right and left visual cortex, when stimulated with blue and yellow through the right eye, which is consistent with right eye color functional ocular dominance (OD) plasticity. Conversely, in female mice, we observed (see Fig 6) a higher SUV in the right and left visual cortex, when stimulated with blue through the left eye, which is consistent with left eye color functional OD plasticity [55]. It was observed that there was greater tracer accumulation in the ocular dominant eye, in male mice in the right eye, and in female mice in the left eye, during stimulation. This may provide support for the anatomic and physiologic basis for OD plasticity, whereby brief deprivation of patterned vision in one eye leads to a reduction in the responses of visual cortical neurons to the deprived eye and a matching increase in responses to the open eye. Although color functional OD has been reported in monkeys [56], and others have reported that short monocular deprivation (4 days) induces a shift in the OD of binocular neurons in the juvenile mouse visual cortex, it is ineffective in adult mice [57]. However, prior studies [55] have underscored that the knowledge of the anatomical basis of cortical OD is essential for understanding OD plasticity following manipulations of visual experience in mice.

In mice and other mammals each eye transmits visual information along the retinogeniculate projection to relay neurons located in the eye-specific layers of the dorsal lateral geniculate nucleus (dLGN). This eye-specific information is in turn relayed to the primary visual cortex (V1) with retinotopic precision via the geniculocortical pathway. In mammals with frontally positioned eyes, contralateral and ipsilateral projections of retinofugal pathways are roughly equal in magnitude [58]. The cortical neurons in binocular visual cortex respond equally to stimulation of either eye [59]. In mice with more laterally placed eyes, there is no obvious correlation between the anatomical and functional representation of the visual field from retina to V1 [60,61]. Only about 3% of the entire retinofugal pathway projects ipsilaterally, and the number of retinal ganglion cells (RGCs) viewing a point in binocular space is nine times greater for the contralateral retina than for the ipsilateral retina [61]. Nonetheless, the relative drive on binocular neurons by the contralateral eye is on average, only two to three times greater than that for the ipsilateral eye [60, 62–65]. Furthermore, even though the number of retinal short-wavelength opsin (S-opsin) and middle to long-wavelength opsin (L-opsin) have been counted [66], the lateral differences in relation to gender have not been studied. Some have suggested that, OD of cortical neurons in mouse V1 results from feed-forward geniculocortical input in a manner similar to mammals with frontally placed eyes [66].

The findings in both male and female mice in the present study are supported by studies performed in other species. It has been demonstrated in anaesthetized cats [67] that, ON (excitatory) and OFF (inhibitory) geniculate neurons with corticopetal axons receive inhibitory inputs from the opposite stream of matching retinotopic location through local circuit. The latter potentiates the ON-OFF dichotomy in a kind of push-pull operation while maintaining spatial specificity [68]. It has been suggested that, the input from ON and OFF streams reach spatially adjoining areas of the brain to generate a receptive field of neurons containing the ON and OFF sub-regions in the primary visual cortex of macaque [69] and cat [70,71].

To illustrate the results in terms of simplified ON-OFF responses, we designated in a matrix of Eye x Visual Cortex x Stimulation for Dark, Light, Blue and Yellow conditions, as ON, OFF or RS (resting state) channels using the result of the *t*-test to determine significant percentage changes from dark stimulus-absent condition (Table A in S1 File). Applying the matrix format, in male mice, the luminance channel dark/light was in RS. This may suggest that, perceptual processes of dark/light contrast, does not impose significant increase or decrease in CMR_Glc_ in male mice. In male mice, the chromatic blue channel evoked the highest significant metabolic response to Blue^ON^ in the left visual cortex through the right eye (BlueR visCtxL^-ON^). While for the chromatic yellow channel the highest response was in the left visual cortex through the right eye (YellowR visCtxL^-ON^). During yellow stimulation, there was concurrent lesser incremental ‘Yellow ^ON^’ effect through the right eye on the ipsilateral right visual cortex (YellowR visCtxR^-ON^). Conversely, there was an inhibitory Yellow^OFF^ effect through the left eye on the contralateral right visual cortex (YellowL visCtxR^-OFF^) and ipsilateral left visual cortex (YellowL visCtxL^-OFF^). The latter shows a heterogeneous pattern of brain regional activations comprising blue/yellow channel ON-OFF regions with stimulation of the visual cortex (BlueR visCtxL^-ON^/YellowR visCtxL^-ON,^ YellowR visCtxR ^-ON^/YellowL visCtxL^-OFF,^ YellowL visCtxR^-OFF^), that is, ‘polychromatic but simple’ model. In male mice, this could be the evidence for push-pull interactions that help to maintain spatial and chromatic opponency in the blue/yellow channel [67].

In female mice, the chromatic Blue^ON^ channel response was highest in the right visual cortex through the left eye (BlueL visCtxR^-ON^). There was concurrent lesser incremental ‘Blue^ON^’ effect through the right eye on the contralateral left visual cortex (BlueR visCtxL^-ON^) and through the left eye on the ipsilateral left visual cortex (BlueL visCtxL^-ON^). In female mice, there is a homogenous monochromatic pattern of brain regional activations of Blue^ON^ (BlueL visCtxR^-ON^, BlueL visCtxL^-ON^ and BlueR visCtxL^-ON^), that is a ‘complex or multiplex’ model. In female mice, this could be evidence of a push-forward interaction observed here. The male and female models of interactions may represent independent gender-related mechanisms that help maintain spatial and chromatic opponency in the blue/yellow channels in mice. The difference in gender-related models may indicate that, in male mice, there was only an intra-hemispheric Blue^ON^ effect in the left visual cortex (BlueR visCtxL^-ON^). While in female mice, there was inter-hemispheric Blue^ON^ effect though predominantly in the right visual cortex (BlueL visCtxR^-ON^) but also activated the left visual cortex (BlueL visCtxL^-ON^ and BlueR visCtxL^-ON^), suggesting that activations of the architectonic areas would be more extensive and complex compared to that in male mice.(Table B in S1 File)

In the present study, the regions of the brain were considered metabolically connected based on whether the estimation of the glucose consumption significantly correlates across subjects in a specific group [72] in cortical networks known to be related to visceral function and mood [73].

Fig 8 demonstrates that, the male brain appear to be optimized for intra-hemispheric pattern, while female mice brain is for inter-hemispheric pattern. There is a correlate in human studies demonstrated with diffusion tensor imaging (DTI), which also showed gender differences of neural networks connecting different regions of the brain, where the male and female brains appeared to be optimized for intra- and inter-hemispheric patterns of communication, respectively [74]. The detailed characteristics of the structures activated by effects of blue color stimulation are provided in the supplementary information (Fig A in S1 File).

In non-primate mammals, the retina contains two classes of cone photopigments that support dichromatic color vision [38]. Experiments using knock-in mice that expressed a human long-wavelength–sensitive (L) cone photopigment in the form of an X-linked polymorphism demonstrated that heterozygous females, whose retinas contained both native mouse pigments and human L pigment, showed enhanced long-wavelength sensitivity and acquired a new capacity for chromatic discrimination [38]. Some of these mice use the extra cone to make trichromatic color discriminations similar to those that are the basis of human color vision. Simultaneous color contrast and color constancy are memory processes associated with color vision. In the present work, simultaneous color contrast was demonstrated as a *prima facie* evidence of color vision in mice, contrary to the preposition by Walls [23]. The ability to discriminate variations in spectral composition must be irrespective of variations in intensity implying color constancy. It was shown that, discrimination was based on chromatic and not intensity related cues. The latter suggests that, the mouse has photoreceptors that contain spectrally distinct photopigments and a nervous system for comparison of the outputs of these receptors in spectrally opponent networks [75, 76]. The observation that the mouse brain can use this information to make spectral discriminations implies that, it has a plastic nervous system that could discriminate between visual stimuli with extensive neural connectivity to other brain regions. Furthermore, in the evolutional trend genetic changes refined the downstream neural circuitry that more efficiently extract color from other sensory information over many generations.

In human studies using advanced neuroimaging techniques, it has been demonstrated that there is gender complementarity of right hemisphere function in males and left hemisphere function in females during color processing [14,17,18], facial processing [77,78] and general intelligence [79–81]. The demonstration of gender-related differences in cerebral asymmetry in mice though opposite to that found in human studies, however, lends support to an earlier proposition based on human studies that, the evolutionary trend of color vision is towards optimization of perception of the ‘whole’ environment by functional coupling of the genes for complementarity of both hemispheres within self, and between both genders [17,18]. Furthermore, the gender complementarity in mice is attuned to adaptive neuroplasticity as observed in human studies [18]. The present work shows novel findings of gender-related cerebral asymmetry for color processing in an animal model, which showed a reverse trend of activation in the left hemisphere in male mice and right hemisphere in female mice. Color is a powerful information channel to the human cognitive system and has been found to play a significant role in enhancing memory performance [82]. Furthermore, it has been suggested that the visual system response to blue light might be a marker for the central nervous system dopamine tone [14].

Clinical observations suggest that, patients with degenerative mental diseases could also have color deficit abnormalities. Alzheimer’s disease patients have unspecific color vision deficiency [83]. Disturbed color vision was found in 54% of patients with endogenous depression and 72% of patients with schizophrenic psychosis [84]. A statistically measurable linkage between color blindness and manic-depressive illness in a family identified by a schizo-affective volunteer has been shown [84]. In Parkinson’s disease there is alteration to color discrimination [85]. The investigation of genetically engineered mice [38, 86, 87] is expected to provide insight into the role of the different genes in the mechanisms of color processing, as well as their roles in degenerative brain diseases.

In conclusion, the present study demonstrates that the effects of blue color on the brain in male mice implements left intra-hemispheric metabolic connectivity, while in female mice, there was right-to-left inter-hemispheric metabolic connectivity with a wide range of brain structures implicated in viscerosensory and visceromotor systems. The present animal model could be used in the study of color processing mechanisms and gender complementarity in normal and pathological brain conditions.

## Supporting information

S1 File Fig A: Schematic diagram of neural connectivity.
Table A: ON/OFF matrix developed from changes observed in SUV in the right and left visual cortex during stimulations in male and female mice.
Table B: Functions of cortical structures activated by blue color stimulation.
Table C: Original data (Standardized Uptake Values).
(PDF)

## Abbreviations

AI: agranular insular cortex
ACC: Anterior cingulate cortex
ACd: dorsal agranular cingulate area
ACv: ventral agranular cingulate areas, dorsal and ventral part
CI: caudal interstitial nucleus of the medial longitudinal fasciculus
CA1: field CA1 of hippocampus
CA2: field CA2 of hippocampus
CA3: field CA3 of hippocampus
Cg1: cingulate area 1
Cg2: cingulated area 2
CIC: central nucleus of the inferior colliculus
CM: central medial thalamic nucleus
CMR_Glc_: cerebral metabolic rate of glucose
CPu: caudate putamen (striatum)
DG: dentate gyrus
DMTg: dorsomedial tegmental area
DP: dorsal peduncular cortex
DTT: dorsal tenia tecta
ECIC: external cortex of the inferior colliculus
GI: granular insular cortex
GrO: granular insular cortex
Gus: gustatory thalamic nucleus
HCNP: hippocampal cholinergic
IL: infralimbic cortex
InC: interstitial nucleus of Cajal
LTDg: laterodorsal tegmental nucleus
LGP (GPe): lateral globus pallidus
LO: lateral orbital frontal cortex
LSI: lateral septal nucleus
M2: secondary motor cortex
MO: medial orbital cortex
MS: medial septal nucleus
OMPFC: orbitomedial prefrontal cortex
PAG: periaqueductal gray
PaS: parasubiculum
Po: posterior thalamic nuclear group
PoDG: polymorph layer of the dentate gyrus
PL: prelimbic cortical area
PrS: presubiculum
R: red nucleus
RI: rostral interstitial nucleus of medial longitudinal fasciculus
S2: secondary somatosensory cortex
SPFPC: subparafascicular thalamic nucleus parvocellular part
VL: ventrolateral thalamic nucleus
VO: ventral orbital cortex
VPL: ventral posterolateral thalamic nucleus
VPM: ventral posteromedial thalamic nucleus
VTT: ventral tenia tecta
