# Supplementary material for: Gender differences in cerebral metabolism for color processing in mice: A PET/MRI Study
Source: PLoS One. 2017 Jul 19;12(7):e0179919. doi: 10.1371/journal.pone.0179919 (PMC5516971; doi:10.1371/journal.pone.0179919)
Supplement: S1 File — Fig A: Schematic diagram of neural connectivity. Table A: ON/OFF matrix developed from changes observed in SUV in the right and left visual cortex during stimulations in male and female mice. Table B: Functions of cortical structures activated by blue color stimulation. Table C: Original data (Standardized Uptake Values). (PDF) [file pone.0179919.s001.pdf]

Supporting Information

ON-OFF response

Assuming that neuronal metabolism is coupled to neuronal activity, it would be expected that, CMR_{Glc} and hence SUV should increase with ON-response, but decrease with OFF-response, and remain similar to dark stimulus-absent condition in resting state (RS) with normal level of brain activity. We designated in a matrix of Eye x Visual Cortex x Stimulation for Dark, Light, Blue and Yellow conditions, as ON, OFF or RS channels using the result of the *t*-test to determine significant percentage changes from dark stimulus-absent condition, replicating (Table 2). The matrix designated statistically significant increase in percentage change as incremental ON-channel (the highest as the main color channel), decrease as decremental OFF-channel, and no change from resting state (RS) in regions of the brain, as shown in Table 2 .

Table A. ON / OFF matrix developed from changes observed in SUV in the right and left visual cortex during stimulations in male and female mice.

Conditions	Eye	Male Mice		Female Mice	
		visCtxR	visCtxL	visCtxR	visCtxL
Dark	R	RS	RS	RS	RS
	L	RS	RS	RS	RS
Light	R	RS	RS	OFF	OFF
	L	RS	RS	RS	RS
Blue	R	RS	ON	RS	ON
	L	RS	RS	ON	ON
Yellow	R	ON	ON	RS	RS
	L	OFF	OFF	RS	RS

R, right eye; L, left eye; visCtx, visual cortex; RS, resting state

In male mice, the luminance channel under dark condition was at rest (RS) in both right and left visual cortex through the right eye and left eye, respectively. Similarly, light condition remained at rest (RS) in both right and left visual cortex through the right eye and left eye. Blue stimulation through the right eye evoked incremental ON-channel in the left visual cortex, while the right visual cortex remained at rest (RS). On the other hand, blue stimulation through the left eye did not alter the resting state (RS) in the right visual cortex and left visual cortex. Yellow stimulation through the right eye evoked incremental ON-channel in the right visual cortex and left visual cortex, respectively. On the other hand, stimulation through the left eye caused a decremental OFF-channel in the right visual cortex and left visual cortex.

In female mice, the luminance channel in dark was at rest (RS) in both the right and left visual cortex through the right eye and left eye, respectively. Conversely, light condition through the right eye caused inhibitory OFF-response in both right and left visual cortex. However, light condition through the left eye did not alter the resting state (RS) in both right and left visual cortex. Blue stimulation through the right eye evoked incremental ON-channel in the left visual cortex, while the right visual cortex remained at rest (RS). On the other hand, blue stimulation through the left eye evoked incremental ON-channel in both the right visual cortex and left visual cortex. Yellow stimulation did not alter the resting state (RS) in the right visual cortex and left visual cortex through the right eye and left eye, respectively.

Gender-related Cortical Networks Activated by Blue Color

Blue color activated in male mice the left, but the right in female mice the area of the orbital and medial prefrontal cortex (OMPFC) with intra- and inter-hemispheric connections to the striatum, medial thalamus and other brain regions, respectively. Each network receives sensory inputs (olfactory, gustatory, visceral afferent, somatic sensory and visual) that appear to be related to feeding. There are also many limbic inputs from the amygdala, entorhinal and

perirhinal cortex, and subiculum. The OMPFC is a complex region containing agranular, dysgranular, and granular regions interact with basal ganglia-thalamic circuit and limbic structures. These structures may also serve as substrate to integrate viscerosensory information with affective signals. The medial network comprise areas on the medial frontal surface together with a few select areas in the orbital cortex, which form the major output from the OMFPC to the hypothalamus and brain stem (especially the periaqueductal gray). The Blue color activation of the medial network as a visceromotor system provides frontal cortical influence over autonomic and endocrine function. The functions of the specific structures are hereby provided in Table 1, and illustrated in the attached Figure S1.

Table B. Functions of cortical structures activated by blue color stimulation

Abbreviation	Full Name	Location and Functional Connectivity
AI	agranular insular cortex	surrounds the rhinal fissure and lacks a granular layer; it is involved in nociceptive [1], autonomic cardiopulmonary [2], and visceral responses to stress [3].
Acd	dorsal anterior cingulate area	belongs prefrontal cortical areas characterized by weak amygdaloid inputs. The Acd are involved with generating rules associated<br>with temporal ordering and motor sequencing of behaviour [4-6].
Acv	ventral anterior cingulate area	the PL, IL, and Acv receive cholinergic inputs from two separate populations of basal forebrain neurons, one innervating all layers and one selectively innervating deep layers. Cholinergic modulation of the medial prefrontal cortex (mPFC) is crucial for various aspects of cognitive and executive behavior, including attention and working memory [6]
CI	caudal interstitial nucleus of the medial longitudinal fasciculus	crossed fiber tracts on each side of the brainstem; main central connection of the oculomotor nerve (III), trochlear nerve (IV) and abducens (VI) and integrates gaze centers (frontal eye field) and head movement (vestibulocochlear nerve II) [7]; involved in saccadic eye movement, vestibulo-ocular and optokinetic reflexes.
CA1	field CA1 of hippocampus	the hippocampal formation consists of six-layered periallocortical regions (the entorhinal cortex, parasubiculum, presubiculum and postsubiculum) and three-layered allocortical regions (the subiculum, Ammon's horn and dentate gyrus). In primates it is located in<br>the medial temporal lobe, below the cortical surface; it contains two main interlocking parts: the hippocampus proper (also called Ammon's horn) and the dentate gyrus; CA1 performs a match–mismatch comparison of memory retrieval with sensory input [8].
CA2	field CA2 of hippocampus	has several features that distinguish it from CA1 and CA3, including a unique gene expression profile, failure to display long-term potentiation and relative resistance to cell death [9]; involved in social memory – the ability of the

		animal to remember another animal of the same species (conspecific) [10].
CA3	field CA3 of hippocampus	located in the medial temporal lobe, lesions of the CA3 and dentate gyrus strongly reduce the enhanced exploration associated with displaced objects, beyond the reduction caused by CA1 lesions [8].
Cg1 and Cg2	cingulate cortex area 1 and area 2	the cingulate cortex, a part of the limbic cortex situated in the medial aspect of the cerebral cortex. It receives inputs from the thalamus and the neocortex, and projects to the entorhinal cortex via the cingulum. It is involved with emotion formation and processing [11], learning and memory [12]. The cingulate cortex area 1 is the rostral part of the anterior cingulate cortex and the frontal area 2 is the caudal and dorsal parts of the anterior cingulate cortex. The anterior cingulate cortex is organized topographically; stimulus attributes predicting reward or no reward are represented in the rostral (Cg1) and ventral (Cg3) parts of the anterior cingulate cortex, while Cg2 (the caudal and dorsal parts) of the anterior cingulate cortex are related to execution of learned instrumental behaviours [13].
CIC	central nucleus of the inferior colliculus	is the major subcortical auditory integration center receiving extrinsic ascending inputs from almost all auditory brainstem nuclei as well as descending inputs from the thalamus and cortex, and intrinsic intracollicular connections for inhibition after hearing onset [14]. Many units in the central nucleus of the inferior colliculus (CIC) respond to amplitude and frequency modulated tones, features found in communication signals [15].
CM	central medial thalamic nucleus	central medial nucleus (CM) is a prominent cell group of the rostral intralaminar nucleus (ILN) of the thalamus. The primary projections of the CM are the anterior and posterior regions of cortex, the claustrum, the caudate-putamen, the nucleus accumbens (ACC), the olfactory tubercle, and the amygdala. The rostral CM (CMr) more strongly targets limbic structures that include medial agranular, anterior cingulate, prelimbic, dorsolateral orbital and dorsal agranular insular cortices, the dorsal striatum, the ACC, and the basolateral nucleus of the amygdala. While the caudal CM (CMc) more heavily projects to sensorimotor cortical structures that include the ventrolateral, lateral and dorsolateral orbital cortices, dorsal, ventral and posterior agranular insular cortices, visceral cortex, primary somatosensory and motor cortices, and perirhinal cortex. The main CMc subcortical projections are to the dorsal striatum and the lateral, central, anterior cortical, and basomedial nuclei of amygdala. The function of CM may be to integrate affective, cognitive and sensorimotor functions for goal-directed behaviour [16].
Cpu	caudate putamen	parts of the basal ganglia which are broadly responsible for sensorimotor coordination, for planned (cognition;

		caudate) and implemented (sensorimotor coordination; putamen) actions [17].
DG	dendate gyrus	the dentate gyrus is the input region of the hippocampus. The cell body of the dentate pyramidal basket cell is located just within the granule cell layer at its border with the polymorphic layer (PoDG). The granule cell layer encloses a cellular region, the PoDG constitutes the third layer of the dentate gyrus. The most prominent cell types located in the PoDG is the mossy cell. The dentate gyrus acts as a preprocessor of incoming information, preparing it for subsequent processing in CA3 [18]. The dentate gyrus receives its major input from the entorhinal cortex, via the so-called perforant pathway. The glutamatergic supramammillary neurons that project to the dentate gyrus also colocalize calretinin; some of these cells also colocalize substance P. The noradrenergic fibers terminate mainly in the polymorphic layer of the dentate gyrus and extend into the stratum lucidum of CA3. A major portion of the fibers of the septal projection from the forebrain to the dentate gyrus are cholinergic. Many of the other septal cells that project to the dentate gyrus are GABAergic [19].
DMTg	dorsomedial tegmental area	the pontine dorsomedial tegmentum appears to participate in regulating the neural mechanism for lordosis [20].
DP	dorsal peduncular cortex	refers to a cytoarchitectonic area on the medial surface of the cerebral hemisphere rostral to the septum, ventral to the infralimbic area and dorsal to the tenia tecta in the mouse [21]. The mid-DP connect to rostro-dorsomedial part of laminae I/II of Vc (rdm-I/II-Vc), periaqueductal gray and solitary tract nucleus, and ipsilaterally in the parabrachial nucleus, trigeminal mesencephalic nucleus, caudal most level of the granular and dysgranular insular cortex (GI/DI). The mid-DP neurons may regulate intraoral and perioral sensory processing (including nociceptive processing) [22].
DTT and VTT	dorsal tenia tecta and ventral tenia tecta	the dorsal (DTT) and ventral tenia tecta (VTT) are the two parts of the tenia tecta that contains four sublayers. High levels of OX_1R mRNA have been detected in tenia tecta. Orexin may have a role in regulation of feeding [23]. They receive direct input from the olfactory tract mitral cells as secondary olfactory structures involved in the discrimination and learning of odor stimuli, and in the production of appropriate behavioral responses [24].
ECIC	external cortex of the inferior colliculus	the inferior colliculus (IC) is a part of midbrain for processing center for monaural and binaural auditory signals[15]. It is sub-divided into the external cortex, lateral cortex, and central cortex. The IC performs the function of integrating multiple audio signals that help to filter out sounds from vocalizing, breathing, and chewing activities.
GI	granular insular cortex	GI is situated just ventral to the secondary somatosensory cortex with a clear granular layer [2]. The integrity of the granular insula is necessary for exhibiting motivation to

		take nicotine and to relapse to nicotine seeking but not for consuming food pellets or to relapse for food seeking [25].
GrO	granule cell layer of the olfactory bulb	Gro is the deepest layer in the olfactory bulb, made up of dendrodendritic granule cells that synapse to the mitral cell layer. GrO receives excitatory glutamate signals from the basal dendrites of the mitral and tufted cells, and in turn releases GABA to cause an inhibitory effect on the mitral cell. Propagated spikes in granule cells also mediate lateral inhibition to other mitral/tufted cells [26].
Gus	gustatory thalamic nucleus	gustatory thalamus is the functional name for the parvicellular region of the ventroposteromedial (VPMpc) nucleus of the thalamus. The VPMpc critical for the preparatory (i.e. food-seeking) rather than the consummatory (i.e. food-eating) aspects of taste-guided behaviour [27].
HCNP	hippocampal cholinergic neurostimulatin g peptide	act cooperatively with nerve growth factor (NGF), to regulate cholinergic phenotype development in the medial septal nucleus [28]. HCNP and its precursor can be a candidate for the key molecules elucidating the underlying association among A-beta, phosphorylated tau, degeneration of dendritic spine and decrease of acetylcholine in Alzheimer brain [29].
IL	infralimbic cortex	located in the ventromedial prefrontal cortex which is important in tonic inhibition of subcortical structures and emotional responses, such as fear. IL regulates the acquisition and expression of behavioral flexibility. IL extensively innervates amygdala nuclei, hypothalamus, most notably including the dorsomedial and lateral hypothalamus [30-32].
InC	interstitial nucleus of Cajal	interstitial nucleus of Cajal (InC) in the midbrain reticular formation regulates the ability to hold eccentric vertical eye position after saccades, phase advance and decreased gain of the vestibule ocular reflex (VOR) induced by sinusoidal vertical rotation. Furthermore, the InC region of alert animals contains many burst-tonic and tonic neurons whose activity is closely correlated with vertical eye movement, not only during spontaneous saccades, but also during VOR, smooth pursuit and optokinetic eye movement [33].
LTDg	laterodorsal tegmental nucleus	neurons in the laterodorsal tegmentum (LDTg) and pedunculopontine tegmental nucleus (PPTg) play important roles in central autonomic circuits of the kidney [34].
LGP (Gpe)	lateral globus pallidus (external Globus Pallidus)	is part of the basal ganglia together with the caudate and putamen. The globus pallidus, is immediately medial to the putamen and has a medial (internal – Gpi) and lateral (external – Gpe) segment. The Gpe is centrally located within the multiple feedback loops of basal ganglia circuits [35]. The output of Gpe is GABAergic, and inhibitory on its targets [36], inputs to Gpe/Gpi can arrive from cerebral cortex via two major distinct pathways, one

		passing through the striatum (Str) and the other through the subthalamic nucleus (STN). Other inputs to Gpe/Gpi originate from the intralaminar thalamic nuclei and brainstem nuclei including the pedunculopontine tegmentum [37]. The Gpe receives a strong glutamatergic projection from the subthalamic nucleus, and both form a coupled pacemaker, which is used as target for deep brain stimulation in Parkinson's disease [38].
LO	lateral orbital frontal cortex	the lateral orbital frontal cortex (OFC) has three sectors: caudal sector has strong connections with the amygdala, midline thalamus, non-isocortical insula and temporal pole; anterior sector has more pronounced connections with the granular insula, association cortex, mediodorsal thalamus, inferior parietal lobule and dorsolateral prefrontal cortex (PFC), involved in higher-order cognition [39]. The lateral OFC is involved in stimulus-outcome associations and the evaluation and possibly reversal of behaviour [40].
LSI	lateral septal nucleus	lateral septal nucleus is divided into major rostral, caudal, and ventral parts. LSN participates in neuroendocrine regulation of the sexual system. It is a chronoregulatory structure which is responsible for the biorhythmologic organization of the functions of the mammalian organism [41].
M2	secondary motor cortex	the secondary motor cortex (M2) is involved in planning of movement. M2 projections target medial/intralaminar thalamic nuclei, which are known to interact with prefrontal areas associated with working memory, perception, and sensory-guided movements. Both layer 5 of the primary and secondary motor cortices projections have many common targets including the basal ganglia, midbrain and medulla. The motor functional roles of M2 may be mainly mediated through layer 6 M2 projections that communicate with frontal areas [42].
MO	medial orbital frontal cortex	medial orbital (MO) and ventral orbital (VO) cortices are prominent divisions of the orbitomedial prefrontal cortex. Distributes the main cortical targets of MO were the orbital, ventral medial prefrontal (mPFC), agranular insular, piriform, retrosplenial, and parahippocampal cortices. The main subcortical targets of MO were the medial striatum, olfactory tubercle, claustrum, nucleus accumbens, septum, substantia innominata, lateral preoptic area, and diagonal band nuclei of the basal forebrain; central, medial, cortical, and basal nuclei of amygdala; paratenial, mediodorsal, and reuniens nuclei of the thalamus; posterior, supramammillary, and lateral nuclei of the hypothalamus; and periaqueductal gray, ventral tegmental area, substantia nigra, dorsal and median raphe, laterodorsal tegmental, and incertus nuclei of the brainstem [43, 44]. The medial OFC is involved in making stimulus-reward associations and with the reinforcement of behavior [40].

MS	medial septal nucleus	the hippocampus receives cholinergic projections from the medial septal nucleus (MS) and Broca's diagonal band that terminate in the CA1, CA3, and dentate gyrus regions [29]. The hippocampal cholinergic neurostimulating peptide (HCNP) induces the synthesis of acetylcholine in the MS [29]. HCNP may be implicated in the underlying association among A-beta, phosphorylated tau, degeneration of dendritic spine and decrease of acetylcholine in Alzheimer brain [29].
PAG (dmPAG)	dorsomedial periaqueductal gray	located in the midbrain, its major functions include analgesia, fear and anxiety, vocalization, lordosis and cardiovascular control [43]. It receives nociceptive afferent neurons from the spinal cord and sends nociceptive projections to thalamic nuclei. It interacts with the amygdala and its lesion alters fear and anxiety produced by stimulation of amygdala. When stimulated it produces vocalization and its lesion produces mutism [44]. The PAG brainstem structures are rich in 5-hydroxytryptamine (5-HT) inputs related to the modulation of pain. The 5-HT2A and 5-HT2C serotonergic receptors in dmPAG and vlPAG columns, plays a critical role in the elaboration of post-ictal antinociception [44].
PaS	parasubiculum	Pas is major input structure of layer 2 of medial entorhinal cortex, where most grid cells are found. It is a prime target of GABAergic and cholinergic medial septal inputs. It receives input from structures that include the subiculum, presubiculum, and anterior thalamus. The PaS might shape entorhinal theta rhythmicity and the (dorsoventral) integration of information across grid scales [45].
PL	prelimbic cortical area	the infralimbic and prelimbic cortices and the lateral prefrontal cortex (i.e. agranular insular cortices), have reciprocal connections with the perirhinal and entorhinal cortex, and with the CA1 field and subiculum of the hippocampal<br>formation. PL region is involved in attentional and response selection functions as well as visual working memory [46].
Po	posterior thalamic nuclear group	consisting of the centre médian and parafascicular nuclei, involved in limbic motor functions [47].
PoDG	polymorphic cell layer of the dentate gyrus	the granule cell layer encloses a cellular region, the polymorphic cell layer, which constitutes the third layer of the dentate gyrus. A number of cell types are located in the polymorphic layer but the most prominent is the mossy cell. Majority of mossy fiber collaterals in the polymorphic cell layer terminate on GABAergic interneurons. Besides the mossy cell, there are a number of fusiform cells in the polymorphic layer. The main difference between the fusiform cell types is whether they have spines or not and the characteristic shapes and sizes of the spines. One

		type the HIPP cell (hilar perforant path-associated cell) are somatostatin-positive cells which colocalize with GABA, and are the source of the somatostatin immunoreactive fibers and terminals in the outer two-thirds of the molecular layer [48].
PrS	presubiculum	presubiculum and parasubiculum are richly interconnected with excitatory synapses. These interconnections can generate giant excitatory synaptic potentials that support the bursting behaviour exhibited by these neurons. Any of the excitatory inputs to deep layer cells can trigger the population bursts and specific inputs from entorhinal cortex produce the after-discharges [49].
R	red nucleus	the red nucleus caudal part is a structure in the midbrain, while the rostral part is of the diencephalon [50]. The occulomotor nerves traverses only in the midbrain part of the red nucleus. The red nucleus sends its axons to the olive (rubro-olivary and reticulo-olivary fibres) and spinal cord (rubrospinal tract). It is pale pink in color; the color is believed to be due to iron, which is present in the red nucleus in at least two different forms: hemoglobin and ferritin. Its functions includes the coordination of muscle tone, body position and gait [50].
RI	rostral interstitial nucleus of medial longitudinal fasciculus	the rostral interstitial nucleus of medial longitudinal fasciculus (RIMLF) is a portion of the medial longitudinal fasciculus which controls vertical gaze. They project to the vestibular nuclei [51].
S2	secondary somatosensory cortex	activated in response to light touch, pain, visceral sensation, and tactile attention [52]
SPFPC	subparafascicular thalamic nucleus parvocellular part	parvocellular subparafascicular thalamic nucleus (SPFPC) is located in the posterior thalamus. The medial SPFPC may process inputs important for sexual behavior, whereas the lateral SPFp may be involved in convergence of auditory and nociceptive inputs important for conditioned fear responses [53].
VL	ventrolateral thalamic nucleus	lesions of the ventrolateral thalamic nucleus strongly hindered the switching of motor activity under the control of the corticospinal tract in rats subjected to section of the rubrospinal tract and lesioning of the red nucleus [54]. There is a role for the VL in sensory processing of synesthesia in which auditory stimuli produced tactile percepts. This suggests that reorganization of thalamocortical axonal connectivity can lead to major changes in perception [55].
VO	ventral orbital prefrontal cortex	medial orbital (MO) and ventral orbital (VO) cortices are prominent divisions of the orbitomedial prefrontal cortex. Distributes to some of these same sites, notably to the striatum, but lacks projections to parts of limbic cortex, to nucleus accumbens, and to the amygdala. VO distributes

		much more strongly, however, than MO to the medial (frontal) agranular, anterior cingulate, sensorimotor, posterior parietal, lateral agranular retrosplenial, and temporal association cortices. VO performs functions such as directed attention [56].
VPL	ventral posterolateral thalamic nucleus	VPL is more important for transmitting visceral nociceptive signals from Brodmann areas 3, 1 and 2 or primary sensorimotor cortex [57].
VPM	ventral posteromedial thalamic nucleus	VPM conveys facial sensory information of the trigeminothalamic tract, from the solitary tract and the trigeminal nerve and projects to the postcentral gyrus. Primary taste afferent inputs is received from the solitary tract and projects to the cortical gustatory area [58].
Hippocampal-Prefrontal Cortex Projections	13a, 13b, 14r, 14c, and Ilm areas	within the hippocampus, the principal projection to the OMPFC arises in the subiculum and terminates in the medial orbital areas 13a, 13b, 14r, 14c, and Ilm. The caudal areas 13a, 13b, and 14c receive the heaviest projection [59].

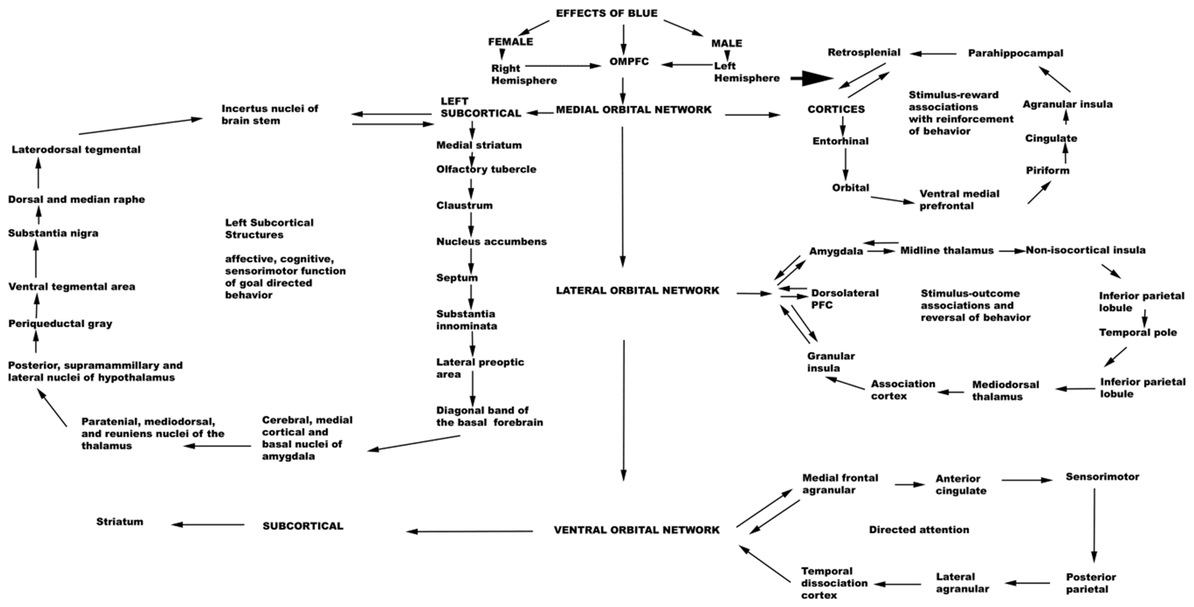

Fig. A Schematic diagram of neural connectivity. The proposed schematic diagram of neural connectivity between the structures of the OMPFC and others during blue stimulation in male and female mice.

References:

1. Burkey AR, Carstens E, Wenniger JJ, Tang J, Jasmin_L. An opioidergic cortical antinociception triggering site in the agranular insular cortex of the rat that contributes to morphine antinociception. J Neurosci. 1996;16: 6612-6623.
2. Cechetto DF, Saper CB. Evidence for a viscerotopic sensory representation in the cortex and thalamus in the rat. J Comp Neurol. 1987;262: 27-45.
3. Krushel LA, van der Kooy D. Visceral cortex: integration of the mucosal senses with limbic information in the rat agranular insular cortex. J Comp Neurol. 1988; 270:39-54, 62-63.
4. Van De Werd HJ, Rajkowska G, Evers P, Uylings HB. Cytoarchitectonic and chemoarchitectonic characterization of the prefrontal cortical areas in the mouse. Brain Struct Funct. 2010; 214: 339–353.
5. Uylings HB, Groenewegen HJ, Kolb B. Do rats have a prefrontal cortex? Behav Brain Res. 2003; 146: 3-17.
6. Bloem B, Schoppink L, Rotaru DC, Faiz A, Hendriks P, Mansvelder HD, et al. Topographic mapping between basal forebrain cholinergic neurons and the medial prefrontal cortex in mice. J Neurosci. 2014;34: 16234-16246.
7. Büttner-Ennever JA, Horn AK, Schmidtke K. Cell groups of the medial longitudinal fasciculus and paramedian tracts. Rev Neurol (Paris). 1989;145: 533-539.
8. Hasselmo ME. The role of hippocampal regions CA3 and CA1 in matching entorhinal input with retrieval of associations between objects and context: theoretical comment on Lee et al. (2005). Behav Neurosci. 2005;119: 342–345.
9. Dudek SM, Alexander GM, Farris S. Rediscovering area CA2: unique properties and functions. Nat Rev Neurosci. 2016;17: 89-102.
10. Hitti FL, Siegelbaum SA. The hippocampal CA2 region is essential for social memory. Nature 2014; 508: 88-92.
11. Hadland KA, Rushworth MF, Gaffan D, Passingham RE. The effect of cingulate lesions on social behaviour and emotion. Neuropsychologia 2003;41: 919–931.
12. Kozlovskiy SA, Vartanov AV, Nikonova EY, Pyasik MM, Velichkovsky BM. The cingulate cortex and human memory processes. Psychology in Russia: State of the Art 2012;5: 231–243.
13. Takenouchi K, Nishijo H, Uwano T, Tamura R, Takigawa M, Ono T. Emotional and behavioral correlates of the anterior cingulate cortex during associative learning in rats. Neuroscience. 1999;93: 1271–1287.
14. Sturm J, Nguyen T, Kandler K. Development of intrinsic connectivity in the central nucleus of the mouse inferior colliculus. J Neurosci. 2014; 34: 15032-15046.
15. Shaddock Palombi P, Backoff PM, Caspary DM. Responses of young and aged rat inferior colliculus neurons to sinusoidally amplitude modulated stimuli. Hear Res. 2001;153: 174-180.
16. Vertes RP, Hoover WB, Rodriguez JJ. Projections of the central medial nucleus of the thalamus in the rat: node in cortical, striatal and limbic forebrain circuitry. Neuroscience. 2012;219: 120–136.
17. Grahn JA, Parkinson JA, Owen AM. The cognitive functions of the caudate nucleus. Prog Neurobiol. 2008; 86: 141-155.
18. Jonas P, Lisman J. Structure, function, and plasticity of hippocampal dentate gyrus microcircuits Front Neural Circuits. 2014;8: 107.
19. Amaral DG, Scharfman HE, Lavenex P. The dentate gyrus: fundamental neuroanatomical organization (dentate gyrus for dummies). Prog Brain Res. 2007;163: 3–22.

20. Yamanouchi K, Nakano Y, Arai Y. Roles of the pontine dorsomedial tegmentum and midbrain central gray in regulating female rat sexual behaviors: Effects of p-chlorophenylalanine. Brain Res Bull. 1990;25: 381–385.

21. Paxinos G, Franklin KBJ. The Mouse Brain in Stereotaxic Coordinates. Second Edition, San Diego: Academic Press; 2001.

22. Akhter F, Haque T, Sato F, Kato T, Ohara H, Fujio T, et al. Projections from the dorsal peduncular cortex to the trigeminal subnucleus caudalis (medullary dorsal horn) and other lower brainstem areas in rats. Neuroscience. 2014;266: 23-37.

23. Trivedi P, Hong Yu, MacNeil DJ, Van der Ploeg LHT,Guan X-M. Distribution of orexin receptor mRNA in the rat brain. FEBS Letters. 1998;438: 71-75.

24. McNamara AM, Cleland TA, Linster C. Characterization of the synaptic properties of olfactory bulb projections. Chem. Senses 2004;29: 225-233.

25. Forget B, Pushparaj A, Le Foll B. Granular insular cortex inactivation as a novel therapeutic strategy for nicotine addiction. Biol Psychiatry. 2010;68: 265-271. doi:10.1016/j.biopsych.2010.01.029.

26. Imai K. Construction of functional neuronal circuitry in the olfactory bulb. Semin Cell Dev Biol. 2014;35: 180–188.

27. Samuelsen CL, Gardner MPH, Fontanini A. Thalamic contribution to cortical processing of taste and expectation. J Neurosci. 2013;33: 1815-1827.

28. Ojika K, Mitake S, Tohdoh N, Appel SH, Otsuka Y, Katada E, et al. Hippocampal cholinergic neurostimulating peptides (HCNP). Prog Neurobiol. 2000;60: 37-83.

29. Matsukawa N, Ojika K. Function of HCNP/HCNP precursor in memory formation. Nihon Shinkei Seishin Yakurigaku Zasshi. 2006;26: 219-227.

30. Cassell MD, Wright DJ. Topography of projections from the medial prefrontal cortex to the amygdala in the rat. Brain Res Bull. 1986;17: 321–333.

31. Mcdonald AJ, Mascagni F, Guo L. Projections of the medial and lateral prefrontal cortices to the amygdala: a *Phaseolus vulgaris* leucoagglutinin study in the rat. Neurosci. 1996;71: 55–75.

32. Hurley KM, Herbert H, Moga MM, Saper CB. Efferent projections of the infralimbic cortex of the rat. J Comp Neurol. 1991;308: 249–276.

33. Fukushima K. The interstitial nucleus of Cajal in the midbrain reticular formation and vertical eye movement. Neurosci Res. 1991;10: 159-187.

34. Ye D, Guo Q, Feng J, Liu C, Yang H, Gao F, et al. Laterodorsal tegmentum and pedunculopontine tegmental nucleus circuits regulate renal functions: Neuroanatomical evidence in mice models. J Huazhong Univ Sci Technolog Med Sci. 2012;32: 216-220.

35. Jaeger D, Kita H. Functional connectivity and integrative properties of globus pallidus neurons. Neuroscience. 2011;198: 44–53.

36. Jessell TM, Emson PC, Paxinos G, Cuello AC. Topographic projections of substance P and GABA pathways in the striato- and pallido-nigral system: a biochemical and immunohistochemical study. Brain Res. 1978;152: 487–498.

37. Deschenes M, Bourassa J, Doan VD, Parent A. A single-cell study of the axonal projections arising from the posterior intralaminar thalamic nuclei in the rat. Eur J Neurosci. 1996;8: 329–343.

38. Chan CS, Glajch KE, Gertler TS, Guzman JN, Mercer JN, Lewis AS, et al. HCN channelopathy in external globus pallidus neurons in models of Parkinson's disease. Nat Neurosci. 2011;14: 85–92.

39. Elliott R, Dolan RJ, Frith CD. Dissociable functions in the medial and lateral orbitofrontal cortex: evidence from human neuroimaging studies. Cereb Cortex. 2000;10: 308–317.

40. Walton ME, Behrens TE, Buckley MJ, Rudebeck PH, Rushworth MF. Separable learning systems in the macaque brain and the role of orbitofrontal cortex in contingent learning. Neuron. 2010; 65: 927–939.

41. Zamorskiĭ II, Myslitskiĭ VF, Pishak VP. The lateral septal nucleus: its morphological and functional organization and its role in the formation of chronorhythms. Usp Fiziol Nauk. 1998; 29: 68-87.

42. Jeong M, Kim Y, Kim J, Ferrante DD, Mitra PP, Osten P, et al. Comparative three-dimensional connectome map of motor cortical projections in the mouse brain. Sci Rep. 2016;6: 20072. doi: 10.1038/srep20072.

43. Behbehani MM. Functional characteristics of the midbrain periaqueductal gray. Prog Neurobiol. 1995;46: 575-605.

44. de Freitas RL, de Oliveira RC, de Oliveira R, Paschoalin-Maurin T, de Aguiar Corrêa FM, Coimbra NC. The role of dorsomedial and ventrolateral columns of the periaqueductal gray matter and in situ 5-HT_2A and 5-HT_2C serotonergic receptors in post-ictal antinociception. Synapse. 2014;68: 16-30.

45. Tang Q, Burgalossi A, Ebbesen CL, Sanfuibetti-Scheck JI, Schmidt H, Tukker JJ, et al. Functional architecture of the rat parasubiculum. J Neurosci. 2016;36: 2289-2301.

46. Sierra-Mercado D, Padilla-Coreano N, Quirk GJ. Dissociable roles of prelimbic and infralimbic cortices, ventral hippocampus, and basolateral amygdala in the expression and extinction of conditioned fear. Neuropsychopharmacology. 2011; 36: 529-538.

47. Van der Werf TD, Witter MP, Groenewegen HJ. The intralaminar and midline nuclei of the thalamus. Anatomical and functional evidence for participation in processes of arousal and awareness. Brain Res Brain Res Rev. 2002;39: 107–140.

48. Amaral DG, Scharfman HE, Lavenex P. The dentate gyrus: fundamental neuroanatomical organization (dentate gyrus for dummies). Prog Brain Res. 2007;163: 3-22.

49. Funahashi M, Stewart M. Presubicular and parasubicular cortical neurons of the rat: functional separation of deep and superficial neurons in vitro. J Physiol. 1997;501: 387-403.

50. Liang H, Paxinos G, Watson C. The red nucleus and the rubrospinal projection in the mouse. Brain Struct Funct. 2012;217: 221–232.

51. Büttner-Ennever JA, Büttner U, Cohen B, Baumgartner G. Vertical glaze paralysis and the rostral interstitial nucleus of the medial longitudinal fasciculus. Brain. 1982;105: 125-149.

52. Eickoff SB, Schleicher A, Zilles K, Amunts K. The human parietal operculum. I. cytoarchitectonic mapping of subdivisions. Cereb Cortex. 2006;16: 254-267.

53. Coolen LM, Veening JG, Petersen DW, Shipley MT. Parvocellular subparafascicular thalamic nucleus in the rat: anatomical and functional compartmentalization. J Comp Neurol. 2003;463: 117–131.

54. Fanardzhyan VV, Papoyan EV, Pogosyan VI, Gevorkyan OV. The role of the ventrolateral nucleus of the thalamus in the switching of descending influences to motor activity in the rat. Neurosci Behav Physiol. 2002;32: 53-59.

55. Ro T, Farnè A, Johnson RM, Wedeen V, Chu Z, Wang ZJ, et al. Feeling sounds after a thalamic lesion. Ann Neurol. 2007;62: 433-441.

56. Hoover WB, Vertes RP. Projections of the medial orbital and ventral orbital cortex in the rat. J Comp Neurol. 2011;519: 3766-3801.

57. Al-Chaer ED, Lawand NB, Westlund KN, Willis WD. Visceral nociceptive input into the ventral posterolateral nucleus of the thalamus: a new function for the dorsal column pathway. J Neurophysiol. 1996;76: 2661-2674.

58. Verhagen JV, Giza BK, Scott TR. Responses to taste stimulation in the ventroposteromedial nucleus of the thalamus in rats. J Neurophysiol. 2003;89: 265-275.

59. Price JL. Prefrontal cortical networks related to visceral function and mood. In: McGinty J, editor. Advancing from the ventral striatum to the extended amygdala. Ann N Y Acad Sci. 1999; 877:383-396.

Table C. Original data (Standardized Uptake Values)

Group	Time (s)	Black Cortex	Black RH	Black LH	LightRTEye Cortex	LightRtEye RH	LightRTEye LH	LightLTEye Cortex	LightLTEye RH	LightLTEye LH
0=female	150	.8880	1.1400	.9940	.7350	.9360	.9700	.9000	1.1340	1.0250
0	150	1.0380	1.3090	1.2160	.8760	.9950	.9200	1.1100	1.1120	1.0570
0	150	.8810	1.0630	1.1050	.8890	1.1540	1.1050	1.1100	1.4040	1.3600
0	150	1.0520	1.3920	1.3740	1.1090	1.1920	1.0750	1.2360	1.5810	1.5960
0	150	.9370	1.2130	1.2530	.8710	1.0610	.9910	.8680	1.1250	1.1800
0	450	.8850	1.1670	1.1580	.7520	.9470	.9890	.8570	1.1250	.9790
0	450	1.0470	1.2600	1.2840	.9800	1.0970	1.0660	1.1830	1.1540	1.1340
0	450	.8760	1.0310	1.1170	.8880	1.1050	1.1400	1.1150	1.4400	1.3790
0	450	1.0670	1.3950	1.3130	1.1830	1.1910	1.2270	1.3030	1.7110	1.7510
0	450	.9040	1.1830	1.1740	.8520	.9670	1.0200	.8410	1.0640	1.1190
0	750	.8820	1.1130	1.1740	.7370	.9180	.9810	.8560	1.1410	.9770
0	750	1.0830	1.3270	1.3340	1.0450	1.1560	1.1450	1.1570	1.1210	1.1140
0	750	.8590	1.0060	1.1020	.9060	1.1620	1.1190	1.1150	1.4360	1.3530
0	750	1.1210	1.4620	1.4850	1.1820	1.1800	1.2410	1.2880	1.7120	1.6260
0	750	.8930	1.1620	1.1360	.8380	.9330	.9810	.8640	1.0820	1.1920
0	1050	.8660	1.1700	1.0450	.7330	.9140	.9810	.8380	1.0400	1.0070
0	1050	1.0880	1.3490	1.3220	1.0800	1.2170	1.1750	1.1510	1.1850	1.0810
0	1050	.8360	.9670	1.0940	.8460	1.1330	1.0570	1.1300	1.4250	1.3910
0	1050	1.0420	1.3700	1.3250	1.1640	1.2100	1.2330	1.2590	1.6770	1.6620
0	1050	.8400	1.1120	1.1400	.7990	.9150	.8800	.8010	1.0340	1.1080
1=male	150	1.0630	1.4830	1.2810	1.3480	1.5830	1.8090	1.0650	1.5330	1.3460
1	150	1.0580	1.4090	1.2250	1.0440	1.1170	1.0430	.8410	.9390	1.0260
1	150	1.2450	1.5210	1.5510	1.3080	1.6310	1.5620	1.2850	1.6650	1.7920
1	150	.8780	.9880	.9710	.6550	.6910	.7160	.8750	1.1400	1.0630
1	150	1.0950	1.3750	1.3360	.8300	1.0520	1.0170	1.1960	1.5050	1.4110
1	450	1.0720	1.4420	1.2000	1.3700	1.7330	1.8350	1.0680	1.4340	1.3910
1	450	1.0450	1.3500	1.2090	1.0370	1.1150	1.0760	.8700	1.0030	1.0660
1	450	1.2740	1.5220	1.7250	1.3420	1.7050	1.6110	1.2900	1.6550	1.7500
1	450	.9440	1.0610	1.0730	.7290	.7990	.7830	.9320	1.1250	1.1440
1	450	1.1640	1.4850	1.4710	.9200	1.1240	1.1490	1.2430	1.5410	1.4860
1	750	1.0900	1.5290	1.3200	1.3870	1.7850	1.6400	1.0580	1.4000	1.4070
1	750	1.0480	1.3760	1.2080	1.0080	1.0760	1.0200	.9190	1.0450	1.1220
1	750	1.2490	1.4540	1.6590	1.3260	1.6980	1.5760	1.2680	1.5790	1.7050
1	750	.9990	1.1180	1.1470	.7550	.7800	.7980	1.0110	1.2390	1.2410
1	750	1.1770	1.4640	1.4540	.9970	1.2660	1.1820	1.2690	1.5020	1.5300
1	1050	1.1180	1.4770	1.4110	1.3640	1.6970	1.7390	1.0300	1.3980	1.3290
1	1050	1.0260	1.2880	1.2440	.9800	1.0200	1.0220	.8910	1.0460	1.0500
1	1050	1.2160	1.5460	1.6080	1.3280	1.7400	1.6330	1.2290	1.6160	1.5530
1	1050	1.0280	1.1880	1.1380	.8310	.8780	.9160	1.0620	1.2960	1.3530
1	1050	1.1710	1.4420	1.3650	1.0280	1.2380	1.3020	1.2490	1.5370	1.4920

Group	Time (s)	BlueRTEyeCortex	BlueRTEyeRH	BlueRTEyeLH	BlueLTEyeCortex	BlueLTEyeRH	BlueLTEyeLH
0=female	150	.9830	1.2300	1.2630	.9890	1.2910	1.2190
0	150	1.1200	1.4360	1.4920	1.1870	1.2510	1.3010
0	150	.8910	1.0460	1.2160	1.1880	1.4070	1.3790
0	150	1.0560	1.2090	1.2370	1.1320	1.3790	1.4010
0	150	1.0440	1.3350	1.2490	1.0300	1.3650	1.1820
0	450	.9040	1.0810	1.1590	1.0210	1.2870	1.2660
0	450	1.1270	1.5180	1.4000	1.1870	1.3030	1.2440
0	450	.9120	1.1420	1.1200	1.2030	1.4420	1.4280

0	450	1.0870	1.2600	1.3680	1.1610	1.4000	1.3480
0	450	1.0690	1.3540	1.3510	.9790	1.3050	1.1270
0	750	.8900	1.0310	1.1390	1.0310	1.2860	1.2840
0	750	1.1600	1.5330	1.5280	1.2100	1.2710	1.2340
0	750	.8890	1.1400	1.2210	1.2500	1.5130	1.5410
0	750	1.1140	1.3370	1.2890	1.1810	1.4290	1.3840
0	750	1.0570	1.3030	1.3320	.9580	1.2600	1.0990
0	1050	.8700	1.0420	1.0500	1.0520	1.3440	1.3360
0	1050	1.0980	1.4360	1.3880	1.2160	1.2940	1.2810
0	1050	.8790	1.1430	1.1070	1.2130	1.4430	1.3890
0	1050	1.0610	1.2890	1.3020	1.1560	1.3630	1.3600
0	1050	1.0780	1.3890	1.3490	.9400	1.1980	1.1230
1=male	150	1.0500	1.4100	1.3730	1.0220	1.3170	1.2890
1	150	1.1270	1.3370	1.4940	.9770	1.2210	1.1210
1	150	1.0950	1.3560	1.5220	.7720	.8770	.9960
1	150	1.2860	1.5750	1.6470	1.1760	1.4300	1.4580
1	150	1.1170	1.3690	1.5200	.9868	1.2113	1.2160
1	450	1.0830	1.4130	1.5010	1.0370	1.3300	1.3140
1	450	1.1240	1.3890	1.5550	.9850	1.2650	1.2810
1	450	1.0970	1.4450	1.4610	.8470	1.0280	1.0550
1	450	1.3180	1.5610	1.6330	1.1620	1.4330	1.3280
1	450	1.1890	1.5590	1.5500	1.0078	1.2640	1.2445
1	750	1.1170	1.5210	1.4340	1.0450	1.3540	1.2780
1	750	1.1260	1.4980	1.4760	.9870	1.1990	1.2270
1	750	1.1510	1.5020	1.4860	.9330	1.1360	1.1670
1	750	1.3340	1.6550	1.5860	1.1610	1.5080	1.3010
1	750	1.2130	1.4530	1.6810	1.0315	1.2993	1.2433
1	1050	1.1160	1.4610	1.4670	1.0550	1.3990	1.2970
1	1050	1.1080	1.3150	1.4720	.9660	1.2660	1.2470
1	1050	1.1100	1.4130	1.4940	.9870	1.2190	1.1810
1	1050	1.3340	1.6540	1.5590	1.1870	1.4400	1.4560
1	1050	1.1980	1.4770	1.6770	1.0186	1.276	1.249

Group	Time (s)	YelRTEyeCortex	YelRTEyeRH	YelRTEyeLH	YelLTEyeCortex	YelLTEyeRH	YelLTEyeLH
0=female	150	.9700	1.2060	1.2400	.9740	1.2750	1.2050
0	150	1.0030	1.3460	1.2320	1.1560	1.3230	1.3920
0	150	.8820	1.1490	1.0000	.8270	1.0080	1.0130
0	150	.9220	1.1580	1.1750	.9640	1.2550	1.2830
0	150	.9220	1.1580	1.1750	.9550	1.2320	1.3220
0	450	.9860	1.2500	1.2670	1.0270	1.3810	1.2820
0	450	1.0170	1.4030	1.2510	1.2030	1.4050	1.4050
0	450	.8950	1.2050	.9840	.8430	1.0750	1.0100
0	450	.9670	1.1880	1.2320	.9710	1.2330	1.2920
0	450	.9670	1.1880	1.2320	.9360	1.2120	1.2880
0	750	.9790	1.2530	1.1860	1.0250	1.2990	1.3460
0	750	1.0460	1.4650	1.2320	1.2560	1.5210	1.5140
0	750	.8980	1.1550	1.0060	.8910	1.0520	1.1330
0	750	.9380	1.1790	1.1870	.9620	1.2680	1.2210
0	750	.9380	1.1790	1.1870	.9450	1.2530	1.3040
0	1050	.9600	1.2730	1.2000	.9820	1.2830	1.2500
0	1050	1.0410	1.4080	1.3390	1.2650	1.4410	1.5310
0	1050	.9410	1.2300	1.0760	.8850	1.1360	1.0900

0	1050	.9330	1.1810	1.1660	.9710	1.2360	1.2750
0	1050	.9330	1.1810	1.1660	.9100	1.2350	1.2010
1=male	150	1.0910	1.4820	1.3110	.9520	1.1610	1.0830
1	150	.9760	1.2410	1.3210	.8590	1.0090	.9140
1	150	1.1340	1.5430	1.4970	1.1490	1.4160	1.4600
1	150	1.1270	1.4770	1.4270	1.0040	1.1860	1.1320
1	150	1.1510	1.4840	1.5520	.9010	1.1560	1.1800
1	450	1.1180	1.5000	1.3560	.9890	1.1970	1.1830
1	450	1.0020	1.2620	1.3700	.9100	1.0660	.9630
1	450	1.1870	1.5740	1.6160	1.1880	1.4940	1.4550
1	450	1.2110	1.4680	1.4470	1.0760	1.2130	1.2690
1	450	1.1580	1.5570	1.4930	.9770	1.2900	1.2430
1	750	1.1030	1.5110	1.2480	1.0420	1.2640	1.2850
1	750	.9930	1.2670	1.4070	.8860	1.0040	.9310
1	750	1.1940	1.6400	1.5970	1.1790	1.4960	1.4700
1	750	1.2130	1.5330	1.4790	1.0970	1.3250	1.2190
1	750	1.1550	1.4600	1.5700	1.0070	1.2890	1.3180
1	1050	1.1490	1.6030	1.3800	1.0280	1.2970	1.2630
1	1050	.9830	1.2140	1.3580	.8770	.9940	.9690
1	1050	1.1980	1.5660	1.5860	1.1810	1.5040	1.4990
1	1050	1.2420	1.5250	1.4440	1.1500	1.3060	1.2900
1	1050	1.1220	1.5150	1.4600	1.0550	1.3960	1.4280
